# Essential amino acids preserve intestinal barrier integrity *via* mitochondrial protection in obesity and gut inflammation

**DOI:** 10.3389/fphar.2025.1694723

**Published:** 2025-12-03

**Authors:** Letizia Spataro, Maurizio Ragni, Agnese Segala, Alice Vetturi, Giulia Sofia Marcotto, Luca Canciani, Michele O. Carruba, Roberto Aquilani, Ginetta Collo, Alessandra Valerio, Enzo Nisoli, Chiara Ruocco

**Affiliations:** 1 Department of Medical Biotechnology and Translational Medicine, Center for Study and Research on Obesity, University of Milan, Milan, Italy; 2 Department of Molecular and Translational Medicine, University of Brescia, Brescia, Italy; 3 Department of Biology and Biotechnology “Lazzaro Spallanzani”, University of Pavia, Pavia, Italy

**Keywords:** essential amino acids, intestinal barrier function, mitochondrial function, gut inflammation, mTOR signaling, leaky gut, intestinal epithelial cells, obesity

## Abstract

**Objective:**

Obesity disrupts intestinal homeostasis, leading to increased permeability (“leaky gut”), mucosal inflammation, and systemic metabolic dysfunction. Mitochondrial impairment in intestinal epithelial cells (IECs) is a central driver of this process. Essential amino acids (EAAs) improve mitochondrial function in metabolic tissues, but their impact on intestinal health remains underexplored. Here, we investigated whether dietary EAAs preserve gut barrier integrity through mitochondrial protection in obesity and inflammation.

**Methods:**

Male C57BL/6N mice were fed a high-fat diet (HFD) or an isocaloric, isonitrogenous EAA-substituted HFD (HFD-EAA) for 33 weeks to assess metabolic outcomes, intestinal barrier function, inflammation, and mitochondrial biogenesis. Parallel, *in vitro* studies in differentiated Caco-2 cells tested an EAA formula enriched with Krebs cycle intermediates (E7), under basal and pro-inflammatory conditions (IL-1β, TNF-α, LPS).

**Results:**

HFD-EAA supplementation prevented and reversed obesity, improved glucose tolerance, reduced mesenteric fat expansion, and preserved intestinal barrier integrity while attenuating inflammation. EAAs restored intestinal length and weight, lowered plasma calprotectin, and normalized citrulline, a biomarker of enterocyte mass. Tight and adherens junction proteins (zonulin-1, occludin, E-cadherin, claudins) were maintained, while pore-forming claudin-2 was reduced. EAAs also upregulated PGC-1α and mitochondrial electron transport chain genes in intestinal epithelial cells (IECs). Their direct effects were confirmed *in vitro* in Caco-2 cells, where E7 increased transepithelial electrical resistance (TEER), enhanced mitochondrial respiration, suppressed inflammation-induced glycolytic reprogramming, activated antioxidant defenses, and reduced IL8 secretion. Mechanistically, E7 promoted eNOS phosphorylation and inhibited mTORC1 signaling.

**Conclusion:**

EAAs protect gut barrier integrity by sustaining mitochondrial biogenesis and function in IECs, thereby reducing obesity- and stress-induced inflammation. These findings highlight EAAs as a promising nutritional strategy to counteract mitochondrial dysfunction and prevent or reverse gut barrier disruption in obesity-related and inflammatory disorders.

## 1 Introduction

In 2021, an estimated 1.00 billion adult males and 1.11 billion adult females were classified as living with overweight or obesity. If historical trends continue, by 2050, the number of adults living with overweight and obesity is projected to reach 3.80 billion, representing over half of the global adult population (Ng et al., 2025). Clinical obesity is a chronic illness characterized by systemic and organ-specific dysfunctions directly induced by excess adiposity (Rubino et al., 2025). In contrast, preclinical obesity refers to a state of excess adiposity with preserved tissue and organ function but an increased risk of progressing to clinical obesity and other non-communicable diseases (Rubino et al., 2025). These dysfunctions involve multiple organ systems, including the liver, skeletal muscle, cardiovascular and central nervous systems, leading to severe, potentially life-threatening complications such as type 2 diabetes mellitus, metabolic dysfunction–associated steatotic liver disease and steatohepatitis, asthma, cardiovascular diseases, cancer, and neurodegenerative disorders (Jin et al., 2023). These pathological consequences are driven by mechanisms such as chronic inflammation, mitochondrial dysfunction, fibrosis, ectopic fat deposition, and mechanical or hemodynamic stress (Reilly and Saltiel, 2017; Xu et al., 2025). In particular, evidences indicate that low-grade, chronic inflammation plays a central role in linking adipose tissue dysfunction to multi-organ impairment, thereby contributing to the wide spectrum of metabolic, cardiovascular, and neurobehavioral complications associated with obesity (Hotamisligil, 2006).

Alteration in gut homeostasis has been implicated as a key contributor to the progression of obesity-related metabolic and inflammatory complications (Cani and Jordan, 2018). Increased intestinal permeability (“leaky gut”) and low-grade mucosal inflammation are key features of obesity, promoting endotoxemia and insulin resistance. Emerging evidence suggests that obesity is also a significant factor in the pathogenesis of inflammatory bowel diseases (IBDs), with pro-inflammatory cytokines derived from adipose tissue exacerbating disease progression (Harper and Zisman, 2016). Experimental studies in mice have shown that obesity worsens colitis pathology (Cheng et al., 2016; Wunderlich et al., 2018). Both diet-induced obesity (DIO) models in mice (Stenman, 2012) and humans with obesity (Genser et al., 2018) show increased intestinal permeability to bacterial products such as lipopolysaccharide (LPS), a potent inflammatory agent. Mouries et al. demonstrated that just 1 week of an obesogenic diet in mice induces dysbiosis, disrupts the gut vascular barrier, and promotes bacterial translocation to the liver, culminating in non-alcoholic steatohepatitis (Mouries et al., 2019).

Mitochondrial dysfunction in intestinal epithelial cells (IECs) plays a key role in driving barrier breakdown, epithelial cell apoptosis, and inflammation in both obesity and IBDs. Guerbette et al. highlighted the differential impact of saturated fatty acids on mitochondrial function, and further described how high-fat diet (HFD) intake leads to metabolic adaptations in IECs (Guerbette et al., 2024). Specifically, excessive lipid consumption reduces mitochondrial number in these cells, impairs their differentiation, and contributes to increased epithelial permeability (Guerbette et al., 2025). Also, DIO exacerbates experimental colitis by elevating oxidative stress and disrupting mitochondrial function, which in turn activates pro-apoptotic pathways in the colon (Li and Li, 2020).

Tight junction (TJ) remodeling and inflammatory cytokine exposure further exacerbate epithelial barrier dysfunction (Ahmad et al., 2017; Bhat et al., 2019; AlMarzooqi et al., 2024). Three main types of cell–cell junctions are known: TJs [occludins, claudins, and zonulin (ZO)], adherens junctions (AJs, such as E-cadherin), and gap junctions. These structures regulate intercellular communication and play distinct roles in tissue homeostasis (AlMarzooqi et al., 2024). Furthermore, serum ZO is a biomarker of impaired intestinal permeability and is associated with diarrhea, dysbiosis, and poor metabolic health. Its levels tend to normalize following sustained weight loss through lifestyle modification or bariatric surgery (Aasbrenn et al., 2020).

Although lifestyle modification and high-protein diets are effective in improving metabolic outcomes (Te Morenga and Mann, 2012; Sanders et al., 2019; Zhu et al., 2020), their impact on intestinal integrity remains poorly defined. In particular, the role of dietary amino acids in modulating epithelial mitochondrial function and gut barrier integrity is incompletely understood. Specific amino acids, including glutamine, arginine, glycine, glutamic acid, and tryptophan, exert local anti-inflammatory effects and support mucosal repair in IBDs (He et al., 2018; Suzuki, 2020; Duanmu et al., 2022; Ji et al., 2023), but studies investigating essential amino acids (EAAs)—the subset required for protein synthesis and metabolic regulation—on intestinal health are scarce. Notably, EAAs stimulate mitochondrial biogenesis and function in metabolic tissues, to improved insulin sensitivity and reduced inflammation (Valerio et al., 2011; Ruocco et al., 2020; 2021; Ragni et al., 2023). These metabolic benefits are largely mediated by increased nitric oxide (NO) production—via endothelial nitric oxide synthase (eNOS) activation—, reduced oxidative stress (Nisoli et al., 2008), and modulation of mechanistic target of rapamycin complex 1 (mTORC1) (D’Antona et al., 2010; Valerio et al., 2011). Yet, whether EAAs can directly preserve intestinal epithelial barrier function, especially in the context of obesity and inflammation, remains unknown.

Here, we tested the hypothesis that dietary EAAs protect gut barrier integrity by preserving mitochondrial function in IECs. We combined an *in vivo* model of DIO with *in vitro* studies using differentiated Caco-2 monolayers under inflammatory stress to (i) assess the impact of EAAs on barrier function, TJ proteins, and epithelial inflammation, and (ii) dissect the mitochondrial and signaling pathways involved. This integrated approach addresses a critical gap linking nutrition, mitochondrial health, and gut barrier integrity in obesity-related gut dysfunction.

## 2 Materials and methods

### 2.1 Animal model of diet-induced obesity and EAA treatment

Male C57BL/6N mice (8 weeks old; Charles River, Calco, Italy) were housed under controlled temperature and humidity with a 12 h light/dark cycle and *ad libitum* access to food and water. After randomization by body weight, mice were fed for 33 weeks with: (i) a HFD (60% fat, 5.2 kcal/g; D12492, Research Diets Inc., New Brunswick, United States; n = 10 mice); (ii) an isocaloric, isonitrogenous, HFD in which casein protein was replaced by an EAA mixture (HFD-EAA; D17073104, Research Diets; n = 10 mice); or (iii) standard chow (3.2 kcal/g; V1534-300, ssniff-Spezialdiäten, Soest, Germany; n = 6 mice) (Supplementary Figure S1). After 25 weeks, a subgroup of obese HFD-fed mice (n = 5) was switched to HFD-EAA for 8 weeks to assess therapeutic effects (HFD > HFD-EAA). The number of mice used for the *in vivo* experiments was calculated taking into account inter-individual variability in HFD responses in C57BL/6N mice (∼18% obesity-resistant) (Boulangé et al., 2013). The dietary EAA content was based on previous studies demonstrating its specificity compared to a control free-amino acid mix matched to casein composition (D’Antona et al., 2010; Ruocco et al., 2020). All diets were irradiated, stored in cool, dry conditions, and used within 6 months. Body weight (BW) and food intake (FI) were recorded weekly. At sacrifice (week 33), mice were euthanized by cervical dislocation. Visceral (epididymal and mesenteric), subcutaneous (inguinal), and brown (interscapular) adipose depots, liver, and intestine, were collected, weighed, and snap-frozen. Intestinal length and wet weight were measured as indirect markers of barrier dysfunction and inflammation. All animal procedures complied with the European Directive 2010/63/EU and Community guidelines and ARRIVE guidelines (Percie du Sert et al., 2020) and were approved by the Italian Ministry of Health (Protocol No. 15/2024-PR).

### 2.2 Glucose homeostasis and insulin sensitivity

Glucose tolerance test (GTT) and insulin tolerance test (ITT) were performed to assess glucose homeostasis and insulin sensitivity. GTT was conducted after 14 and 31 weeks of treatment (*i.e.*, 6 weeks after diet shift in the HFD > HFD-EAA group) following an overnight fast (16–18 h). Mice received an intraperitoneal (i.p.) glucose bolus (1.5 g/kg BW; #G6152, Sigma Aldrich, Milan, Italy). ITT was performed after 4 h daytime fast, with i.p. injection of insulin (0.75 U/kg BW; Apidra, Sanofi, Milan, Italy). Blood glucose was measured from tail-vein at 0, 15, 30, 60, and 120 min using a glucometer (OneTouch Verio Reflect, LifeScan, Sesto San Giovanni, Italy) with corresponding strips. Glucose tolerance and insulin sensitivity were quantified by calculating the area under the curve (AUC) using the trapezoid method (Ruocco et al., 2022).

### 2.3 Faecal output, energy absorption and digestive efficiency

At week 27, mice were individually housed for 48 h with *ad libitum* access to their assigned diets to assess faecal output. FI (g) and faecal mass (g) were measured, and the percentage of food excreted was calculated as faecal weight/FI x 100. At week 33, faecal energy content (kJ/kg) and elemental composition (carbon, nitrogen, hydrogen; % dry mass) were determined by bomb calorimetry (LabAnalysis Group, Casanova Lonati, Pavia, Italy). Energy intake (kJ) was calculated as FI (g) x diet caloric density (kcal/g) x 4.184 (kcal to kJ). Faecal energy excretion (kJ) was determined as faecal weight (g) x faecal energy content (kJ/kg)/1,000. Digestive efficiency (%) was calculated as digestive efficiency (index of intestinal energy absorption) = [1 – (energy excreted/energy ingested)] × 100 (Meyer et al., 2009).

### 2.4 Plasma profiling of amino acids and calprotectin

At the end of treatment, blood was collected *via* submandibular venipuncture into EDTA-treated tubes (3 mM; Sigma Aldrich, Merck Life Science, Milan, Italy) and centrifuged (8,000 × g, 10 min, 4 °C) to obtain plasma. *Amino acid profiling*. Circulating amino acids were quantified by cation-exchange chromatography with post-column ninhydrin derivatization using a Biochrom 30+ Amino Acid Analyzer (Biochrom Ltd, ERRECI S.r.l, Pieve Emanuele, Milan, Italy). Briefly, plasma was mixed 1:1 with 5% sulphosalicylic acid containing L-norleucine (250 mM) as an internal standard, incubated for 30 min at 4 °C, and centrifuged (10,000 × g, 5 min, 4 °C). Supernatants were filtered (0.22 μm) and analyzed against calibration standards (250 mM for each amino acid). Amino acids were detected at 440 and 570 nm after ninhydrin derivatization (135 °C) and quantified spectrophotometrically (Ragni et al., 2022). *Calprotectin*. Plasma calprotectin levels were measured using Mouse S100A8/S100A9 ELISA Kit (#EM67RB; Invitrogen, Thermo Fisher, Monza, MI, Italy), following the manufacturer’s instructions.

### 2.5 Caco-2 cells and *in vitro* model of gut inflammation

#### 2.5.1 Cell culture and differentiation

Human colon adenocarcinoma Caco-2 cells (ATCC-HTB-37, LGC, Milan, Italy) were cultured in Eagle’s Minimum Essential Medium (EMEM; ATCC 30-2003) supplemented with 10% fetal bovine serum (FBS; ATCC 30-2020), 100 U/mL penicillin, and 100 μg/mL streptomycin (Euroclone, Milan, Italy) at 37 °C in 5% CO_2_. Cells were seeded at passages 10–20 and allowed to differentiate for 14–21 days post-confluence, forming polarized monolayers with TJs and AJs and brush borders (Wang et al., 2011).

#### 2.5.2 Amino acid mixture treatments

Differentiated Caco-2 cells were treated with an EAA mixture enriched with Krebs cycle intermediates—citric, malic, and succinic acids—referred to as E7 (0.1% or 1.0% w/v) for 24 or 48 h to assess dose- and time-dependent effects (Supplementary Figure S2). A standard EAA mixture (without Krebs cycle intermediates) was used for comparison (1.0% w/v; Supplementary Figure S2). Concentrations were selected based on previous studies (D’Antona et al., 2010; Ruocco et al., 2020; Tedesco et al., 2020a).

#### 2.5.3 Inflammatory model

To model gut inflammation, differentiated Caco-2 cells (day 14) were pre-treated with E7 (1.0%, 1 h) (Hollebeeck et al., 2012), followed by a 24–72 h exposure to a mixture of inflammatory stimuli (IS), including IL-1β (25 ng/mL), TNF-α (50 ng/mL), and LPS (10 μg/mL) in EMEM with 1% heat-inactivated FBS (Van De Walle et al., 2008; 2010) (Supplementary Figures S2A–C).

#### 2.5.4 mTOR pathway inhibition

To assess pathway involvement, cells were pre-treated with rapamycin (50 nM, Sigma Aldrich) for 1 h, with or without E7, followed by IS exposure for 48 h (Tedesco et al., 2022).

#### 2.5.5 Mitochondrial function inhibition

Differentiated Caco-2 cells (14 days) were pre-treated with E7 (1.0%) for 24 h and subsequently exposed to antimycin A (0.1–1.0 µM; Sigma-Aldrich), a selective complex III inhibitor, for 24 h. Cell viability was assessed by MTT (Crakes et al., 2019).

### 2.6 Cell vitality assay

Caco-2 cell viability was assessed using two complementary methods, the MTT [3-(4,5-dimethylthiazol-2-yl)-2,5-diphenyltetrazolium bromide] assay (Sigma Aldrich) and the Sulforhodamine B (SRB)-based *In Vitro* Toxicology Assay Kit (TOX6, Sigma Aldrich). For the MTT assay, differentiated Caco-2 cells were seeded 75,000 cells/well in 96-well plates (100 µL/wells) and treated with E7 (0.1% or 1.0%) with or without IS for 24, 48, or 72 h. At the end of the treatment, MTT (5 mg/mL in PBS; 20 μL) was added and incubated for 4 h. Formazan crystals were dissolved overnight in 5% SDS/0.1 M HCl (100 μL/well) at 37 °C, and absorbance was measured at 570 nm/655 nm using a microplate reader (Ragni et al., 2022). Alternatively, the SRB-based TOX6 assay was performed according to the manufacturer’s instructions to confirm cell viability.

### 2.7 Gene expression analysis

Total RNA was isolated from jejunal samples or differentiated Caco-2 cells (seeded 5 × 10^5^ cells/well; 24 well plates) using the RNeasy Mini Kit (Qiagen, Milan, Italy) and treated with DNase according to the manufacturer’s protocol (Bio-Rad Laboratories, Milan, Italy). cDNA was synthesized from 1 µg of RNA using the iScript cDNA Synthesis Kit (Bio-Rad Laboratories). Quantitative real-time PCR (qRT-PCR) was performed with iTaq Universal SYBR Green SuperMix (BioRad Laboratories) on a CFX Connect Real-Time PCR System (Bio-Rad Laboratories). Primer sequences are listed in Supplementary Table S1, designed using Primer3 software (version 4.1.0). Gene expression was normalized to GAPDH using the ΔΔCt method. Relative expression was calculated as 2^−ΔΔCT^, where ΔΔCt represents the difference between ΔCt of each sample and ΔCt of the control group (Corsetti et al., 2014).

### 2.8 Immunoblot analysis

Protein extracts were prepared from differentiated Caco-2 cells (seeded 1.5 × 10^6^ cells/well; 6-well plates) using Mammalian Protein Extraction Reagent (M-PER, #78501, Pierce, Thermo Fisher Scientific, Merck, Milan, Italy), supplemented with protease and phosphatase inhibitors (#PPC1010, Sigma-Aldrich). Protein concentration was determined using the bicinchoninic acid (BCA) assay (#EMP014500, Euroclone). Equal amounts of protein were resolved by SDS-PAGE under reducing conditions and transferred to nitrocellulose or polyvinylidene difluoride (PVDF) membrane (#1704158 and #1704156, Bio-Rad Laboratories). Membranes were incubated with primary antibodies against: ZO-1 (#40-2300; Invitrogen, Thermo Fisher Scientific), Occludin (ab216327, Abcam, Prodotti Gianni, Milan, Italy), peroxisome proliferator-activated receptor γ coactivator 1α (PGC-1α, ab191838, Abcam), cytochrome c oxidase subunit IV (COX-IV, #4844, Cell Signaling Technology, Euroclone), phospho-eNOS (Ser1177, #9571), total eNOS (#9572), phospho-p70S6K (Thr389, #9205), total p70S6K (#9202), phospho-S6 (Ser235/236, # 4858), total S6 (# 2217) (all Cell Signaling Technology, Euroclone). Antibody dilutions ranged from 1:500 to 1:1,000; vinculin (1:3,000; V9131, Sigma-Aldrich) served as loading control. After visualization of phosphorylated proteins, membranes were stripped (Restore™ Stripping Buffer, #EMP100500, Euroclone) and reprobed for total proteins. Detection was performed with HRP-conjugated secondary antibodies (Cell Signaling Technology) and SuperSignal Substrate (#EMP011005, Euroclone) (Corsetti et al., 2014). Bands were imaged with Chemidoc XRS+ (Bio-Rad Laboratories) and quantified by ImageLab software 6.1 (BioRad Laboratories).

### 2.9 Evaluation of intestinal barrier integrity *in vitro*

Caco-2 cells were seeded on Transwell® polycarbonate membrane inserts (0.4 µm pore size; #CLS3470-48EA, Sigma-Aldrich) at a density of 1 × 10^5^ cells/well (24 well plates). After 14 days of differentiation to form polarized monolayers, barrier integrity was evaluated under both basal and inflammatory conditions. *Basal conditions*. Cells were treated apically with E7 (0.1% or 1.0%) for 24 or 48 h. *Inflammatory conditions*. Cells were pre-treated apically with E7 (1.0%) for 24 h, then exposed to IS consisting of IL-1β (25 ng/mL) and TNF-α (100 ng/mL) added to the basolateral side, and LPS (5 μg/mL) added to both apical and basolateral compartments, for 24, 48 or 72 h (Van De Walle et al., 2010; Varasteh et al., 2018). *Mitochondrial function inhibition*. Cells were apically pre-treated with E7 (1.0%) for 24 h, followed by antimycin A (0.1–1.0 µM) for 24 h. *Barrier assessment*. Transepithelial electrical resistance (TEER) was measured using an EVOM3 epithelial volt/Ω m (World Precision Instruments, MatTek *In Vitro* Life Science Laboratories, Slovak Republic) at indicated time points. TEER values were expressed as % relative to untreated control (Hiebl et al., 2020).

### 2.10 Mitochondrial respiration analysis

#### 2.10.1 Clark electrode

Basal oxygen consumption rate (OCR) was measured in differentiated Caco-2 cells using a Clark-type oxygen electrode (Rank Brothers Ltd., Newbury, UK). Cells were seeded at 2 × 10^6^ in a 100 mm² dish and, upon reaching differentiation (14 days), were treated with E7 (1.0%) for 48 h. Cells were then harvested, resuspended in EMEM (1% heat-inactivated FBS, 100 U/mL penicillin, and 100 μg/mL streptomycin), and transferred to the respiration chamber at 37 °C. Sequential additions included oligomycin (0.01 mg/mL; #O4876 Sigma-Aldrich) to inhibit ATP synthase, carbonyl cyanide 4-(trifluoromethoxy)phenylhydrazone (FCCP, 500 nM; #C2920 Sigma-Aldrich) to assess maximal uncoupled respiration, and rotenone/antimycin (500 nM each; #R8875/#A8674 Sigma-Aldrich). OCR was normalized to total protein content (BCA assay).

#### 2.10.2 Seahorse XF

Mitochondrial respiration and glycolysis were assessed in the inflammatory model using a Seahorse XFe24 Extracellular Flux Analyzer (Agilent, Santa Clara, CA, United States). Caco-2 cells (25,000 cells/well) were seeded in Seahorse XFe24 V7 PS Cell Culture Microplates (#100777-004, Agilent) and cultured for 48 h. Cells were then pre-treated with E7 (1.0%) for 1 h, followed by exposure to IS for an additional 24 h (Crakes et al., 2019). On the day of assay, cells were incubated in Seahorse XF DMEM Medium (pH 7.4; #103575-100, Agilent) containing 5.5 mM glucose (#G8270, Sigma-Aldrich, Milan, Italy), 2 mM L-glutamine (#G7513, Sigma-Aldrich), and 1 mM sodium pyruvate (#S8636, Sigma-Aldrich) for 1 h. OCR and extracellular acidification rate (ECAR) were measured using the Seahorse XF T Cell Metabolic Profiling Kit (#103772-100, Agilent), following sequential injections: oligomycin A (1.5 µM; for proton leak), N5,N6-bis(2-Fluorophenyl)[1,2,5]oxadiazolo[3,4-b]pyrazine-5,6-diamine] (BAM15, 2.5 µM; uncoupler for maximal respiration), and rotenone/antimycin A (0.5 µM each; mitochondrial respiration inhibitors) (Ruocco et al., 2020). Data were normalized to DNA content (CyQUANT™ Cell Proliferation Assay, #C7026, Thermo Fisher Scientific) and analyzed using Wave software (Agilent).

### 2.11 Immunofluorescence staining and image analysis

Caco-2 cells (300,000 cells/well) were seeded on poly-L-lysine-precoated glass coverslips in a 24-well plates. After 14 days of differentiation, cells were pre-treated with E7 (1.0%) and exposed to IS for 48 h. Cells were then fixed with cold methanol (100%, −20 °C, 10 min), blocked with 3% BSA (#A2153, Sigma-Aldrich) and 1% normal goat serum (NGS, #5425, Cell Signaling Technology) in PBS for 1 h at room temperature, and incubated overnight at 4 °C with anti-occludin antibody (#ab216327, 1:250) in PBS 0.2% BSA and 1.0% NGS. After washing, cells were incubated with Alexa Fluor® 488-conjugated goat anti-rabbit IgG (1:1,000; #111-485-144, Jackson ImmunoResearch, Euroclone) in PBS with 0.1% Triton X-100 (#T-8787, Sigma-Aldrich) for 1 h at room temperature, and nuclei were counterstained with 4′,6-diamidino-2-phenylindole (DAPI; 1:1,500, D1306, Molecular Probes-Invitrogen) in PBS. Images were acquired using a Zeiss Axio Observer Apotome three microscope (×63 oil immersion objective; 2752 × 2208 pixels). At least 25 random fields per condition were analyzed. Uniform thresholding was applied (Zen Lite v3.11 software, Zeiss) to remove background. Occludin-positive fluorescence areas and nuclei counts were quantified with ImageJ (Fiji).

### 2.12 IL8 secretion in cell culture supernatants

Differentiated Caco-2 cells (14 days; 24-well plate) were pre-treated with E7 (1.0%) and subsequently exposed to IS for 24, 48, or 72 h. At each time point, culture supernatants (500 µL/well) were collected and centrifuged (2,000 × g, 10 min) to remove debris. IL8 concentrations were measured using the Human IL8 ELISA Kit (ab214030, Abcam) following the manufacturer’s instructions and expressed as pg/mL.

### 2.13 Statistical analysis

Data are expressed as mean ± SEM, with n indicating independent biological replicates (see figure legends). Outlier analysis was performed using the ROUT method (*Q* = 1%). Normality of data distribution was assessed using multiple tests (D’Agostino–Pearson, Anderson–Darling, Shapiro–Wilk, and Kolmogorov–Smirnov). The homogeneity of variances was verified using Brown–Forsythe and Bartlett’s tests. For data that met the assumption of normality, parametric tests were used to assess statistical significance. Comparisons between two groups were conducted using an unpaired Student’s t-test, while one-way or two-way ANOVA followed by Tukey’s *post hoc* test was applied for multiple comparisons. When data did not meet the assumption of normality, non-parametric tests were employed. Comparisons between two groups were performed using the Mann–Whitney test, and the Kruskal–Wallis test followed by Dunn’s *post hoc* test was applied for multiple comparisons. Significance was set at p < 0.05. Statistical analyses were performed using the Prism 6.0 software (GraphPad Software, Inc.).

## 3 Results

### 3.1 EAA diet prevents and reverts obesity and type 2 diabetes in DIO mice

**FIGURE 1:**
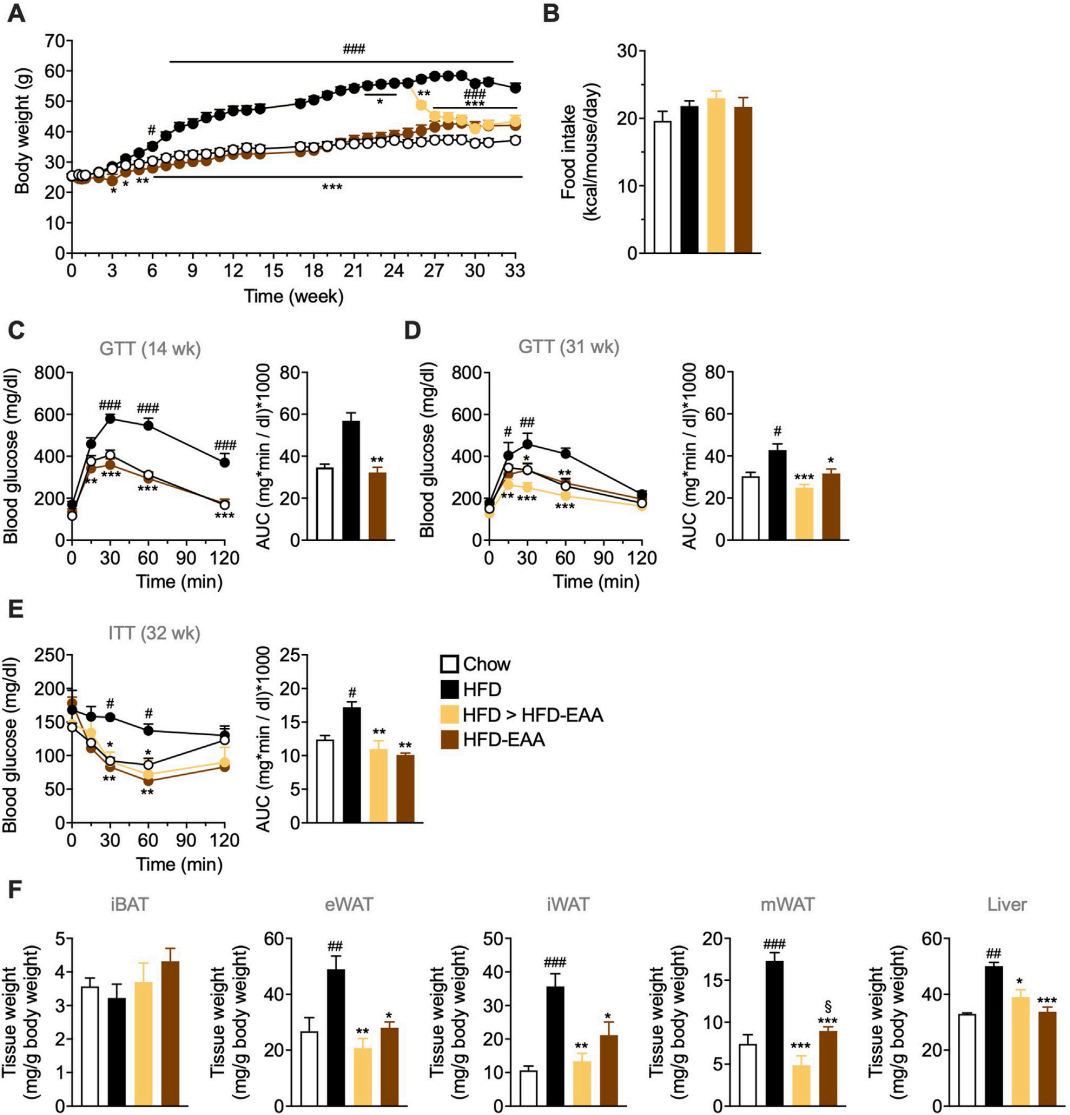
EAA-based diet consumption prevents and reverts obesity and glucose intolerance in diet-induced obese mice. **(A)** Body weight (BW, g). **(B)** Cumulative food intake (kcal/mouse/day). **(C, D)** Glucose tolerance test (GTT): glucose (1.5 g/kg BW; i.p.) administered after overnight fasting at the indicated time points. **(E)** Insulin tolerance test (ITT): insulin (0.75 U/kg BW; i.p.) administered after 4 h of fasting. Blood glucose was measured from tail-vein blood. Results are reported as time-course curves (*left*) and area under the curve (AUC) (*right*). **(F)** Weight of interscapular brown adipose tissue (iBAT), epididymal white adipose tissue (eWAT), inguinal white adipose tissue (iWAT), mesenteric white adipose tissue (mWAT), and liver, expressed as mg per g BW. Data are mean ± SEM (**A, B, F** n = 6–10/group; **C** n = 6–7/group; **D, E** n = 5/group). Two-way ANOVA (**A, C–E** left) or one-way ANOVA (**B, D and E** right, **(F)** Kruskal–Wallis test (**C** right). #p < 0.05, ##p < 0.01, ###p < 0.001 vs. Chow; *p < 0.05, **p < 0.01, ***p < 0.001 vs. HFD; §p < 0.05 vs. HFD > HFD-EAA.

To assess the preventive and therapeutic effects of EAAs in DIO, male C57BL/6N mice were fed HFD or HFD-EAA for 33 weeks, with a subgroup of obese HFD-fed mice switched to HFD-EAA after 25 weeks (HFD > HFD-EAA). HFD feeding induced progressive weight gain (+47% vs. chow), with no substantial differences in FI (Figures 1A,B). In contrast, HFD-EAA diet significantly reduced BW both in the preventive (−22% vs. HFD) and therapeutic (−23% vs. HFD) settings, despite similar caloric intake (Figures 1A,B). Furthermore, HFD feeding impaired glucose tolerance and insulin sensitivity and promoted the accumulation of visceral (epididymal and mesenteric) and subcutaneous (iWAT) fat, accompanied by increased liver weight (Figures 1C–F). Strikingly, HFD-EAA both prevented and reversed these metabolic disturbances, concomitant with reduced adipose depots and decreased liver weight (Figures 1C–F). Notably, mesenteric fat reduction is particularly relevant because visceral adiposity contributes to systemic low-grade inflammation and intestinal barrier dysfunction (Kredel and Siegmund, 2014). Mesenteric fat expansion (“creeping fat”) is a hallmark of Crohn’s disease and aggravates intestinal inflammation (Bryant et al., 2019; Ha et al., 2020). Consistent with this, EAA-mediated reduction of visceral fat mass has been linked to improved metabolic and intestinal outcomes (Luck et al., 2015). In summary, HFD-EAA both prevented and reversed obesity-associated metabolic disturbances, reduced visceral adiposity—including mesenteric fat—and improved glucose homeostasis, establishing a favorable systemic context for intestinal barrier protection.

### 3.2 EAA diet modulates nutrient gut absorption favoring amino acid transport

**FIGURE 2:**
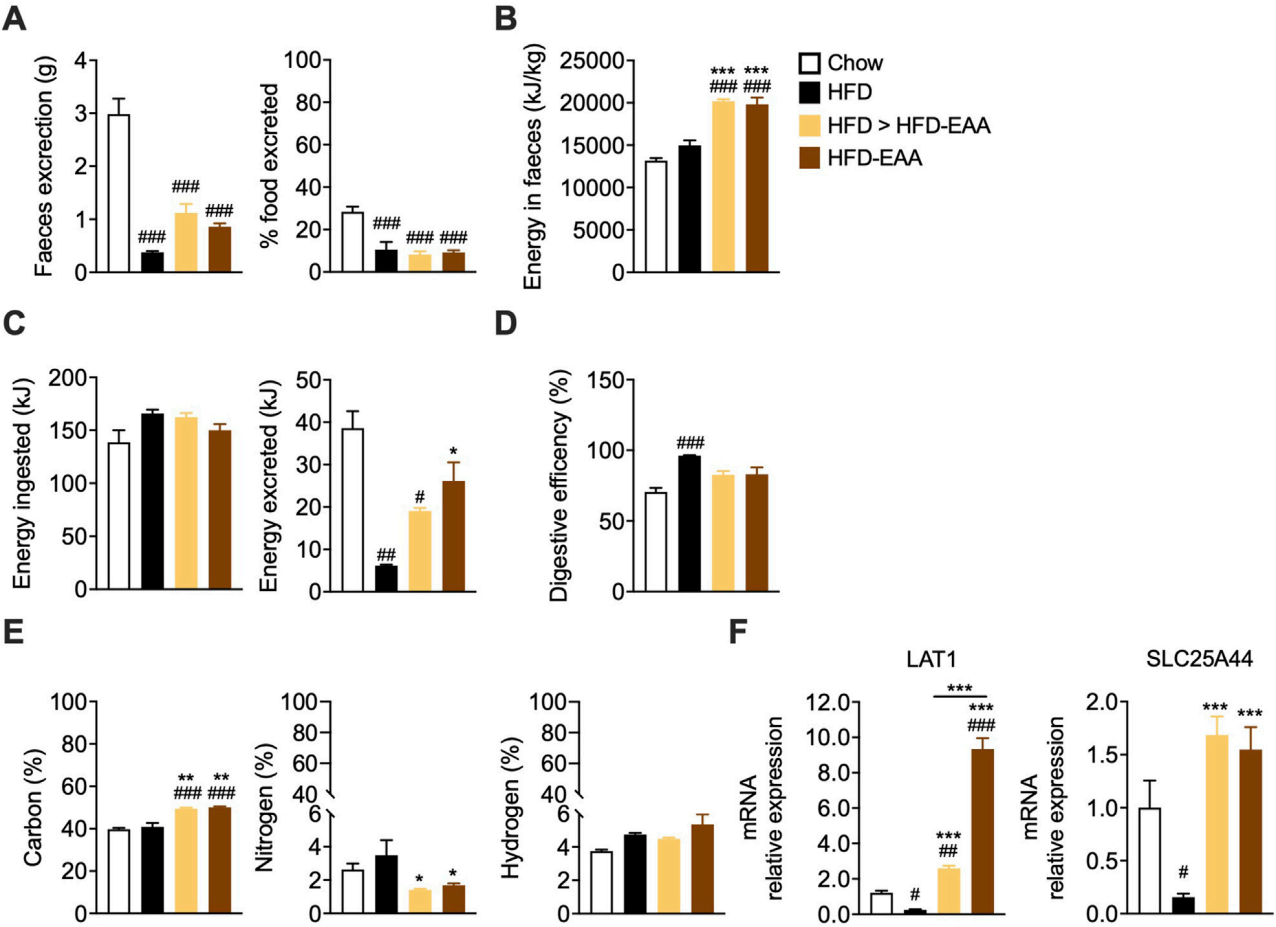
EAA-based diet modulates nutrient absorption and enhances amino acid transport. **(A)** Faecal weight (g; *left*) and percentage of food excreted (%; *right*), calculated as faecal weight (g)/food intake (g) × 100, measured in mice fed *ad libitum* for 48 h, at week 27 of treatment. **(B)** Faecal energy content (kJ/kg), assessed by bomb calorimetry at week 33. **(C)** Energy intake (kJ; *left*), calculated as food intake (g) × dietary energy density (kcal/g) × 4.184 (kcal to kJ conversion), and faecal energy excreted (kJ; *right*), calculated as faecal weight (g) × faecal energy content (kJ/kg) ÷ 1,000 at week 33. **(D)** Digestive efficiency (%), calculated as [1 – (energy excreted/energy ingested)] × 100 (week 33). **(E)** Carbon, nitrogen, and hydrogen content in faeces (% dry mass), determined by bomb calorimetry (week 33). **(F)** mRNA expression of amino acid transporters in the jejunum. Transcript levels were normalized to GAPDH and expressed relative to chow-fed mice (set as 1.0). Data are mean ± SEM (**A**: n = 6–10/group; **B–E**: n = 3/group; **F** n = 3–4/group). One-way ANOVA **(A–F)**. Kruskal–Wallis test (**E** right). #p < 0.05, ##p < 0.01, ###p < 0.001 vs. Chow; *p < 0.05, **p < 0.01, ***p < 0.001 vs. HFD.

We next examined how EAA supplementation influenced nutrient handling in DIO. Consistent with previous findings in low-fat contexts (Ruocco et al., 2020), HFD and HFD-EAA reduced faecal output and the percentage of excreted food compared to chow (Figure 2A), indicating slower intestinal transit, a feature often associated with obesity-related gut dysfunction. However, when evaluating caloric absorption using bomb calorimetry—a measure of digestive function and caloric bioavailability—a different pattern emerged. EAA treatment increased the faecal energy content, which remained unchanged in the HFD group compared to the chow group (Figure 2B). This suggests that, despite the reduced faecal output, HFD-fed mice do not exhibit greater energy loss, indicating a potentially non-selective and passive nutrient absorption likely due to increased intestinal permeability (*i.e.*, leaky gut). Accordingly, despite similar FI, the amount of energy excreted in faeces was lower, and the absorption efficiency (*i.e.*, retained energy relative to ingested food) was higher in HFD-fed mice (Figures 2C,D). This effect may reflect a compensatory metabolic adaptation or result from increased paracellular transport due to gut barrier dysfunction (Murphy et al., 2015). In contrast, EAA-treated mice exhibited greater faecal energy loss and similar digestive efficiency compare to chow (Figures 2C,D). This suggests a selective modulation of nutrient absorption without impairing overall intestinal function. Elemental analysis of faeces supports this interpretation. EAA diets led to a higher faecal carbon content, indicating reduced absorption of carbohydrates and/or lipids, and a significant decrease in faecal nitrogen, consistent with improved protein/amino acid absorption (Figure 2E). Gene expression analysis aligns with these findings. HFD downregulated LAT1, the L-type amino acid transporter 1, a key amino acid transporter in IECs (Fraga et al., 2005), whereas HFD-EAA restored its expression, suggesting a likely increased intestinal EAA absorption following supplementation (Figure 2F). Similarly, HFD reduced SLC25A44, a mitochondrial amino acid transporter (Yoneshiro et al., 2019), which was normalized by EAAs, suggesting preserved mitochondrial integrity and amino acid utilization (markers of improved gut and metabolic efficiency) (Figure 2F). Plasma profiling confirmed increased levels of EAAs included in HFD-EAA diet (Supplementary Figure S3), while correlation analysis linked amino acids such as asparagine, phenylalanine, and tyrosine to impaired glucose homeostasis and visceral adiposity, both normalized by EAAs (Supplementary Table S2). In addition, arginine, histidine, lysine, and tryptophan correlated with faecal output, potentially indicating differences in absorption efficiency (Supplementary Table S2). Collectively, EAAs selectively improved amino acid absorption and utilization, contrasting with the non-specific nutrient uptake seen in HFD-fed mice. This effect likely contributes to enhanced epithelial mitochondrial function and intestinal barrier integrity.

### 3.3 EAAs preserve intestinal barrier integrity and reduce inflammation *via* mitochondrial pathways

**FIGURE 3:**
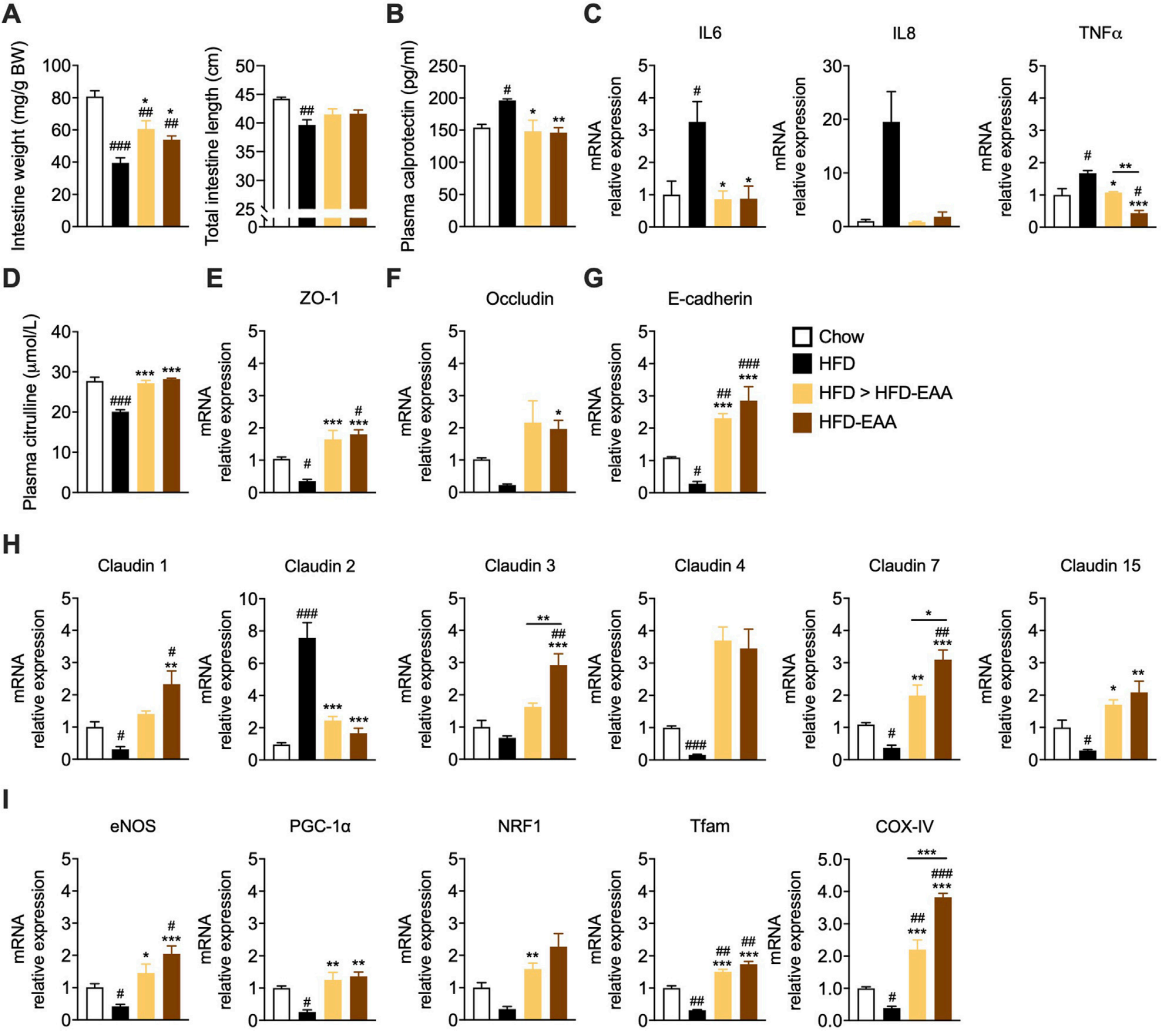
EAA-based diet preserves intestinal barrier integrity and reduces inflammation *via* mitochondrial pathways. **(A)** Intestine weight normalized to BW (mg/g; *left*) and length (cm; *right*). **(B)** Plasma calprotectin levels (pg/mL) at week 33, as a marker of inflammation. **(C and E–I)** mRNA expression in jejunum at week 33. Inflammatory markers (IL6, IL8, TNFα) **(C)**. Intestinal barrier markers (ZO-1, occludin, E-cadherin, claudins) **(E–H)**. Mitochondrial biogenesis markers **(I)**. Transcript levels were normalized to GAPDH and expressed relative to control (CTRL) mice (set as 1.0). **(D)** Plasma citrulline levels (µmol/L) at week 33, as biomarker of gut permeability and enterocyte mass. Data are mean ± SEM (**A**: n = 6–10/group; **B** n = 4–6/group; **C and E–I**: n = 3–4/group; **D** n = 3–6/group). One-way ANOVA **(A–I)** or Kruskal–Wallis test (**C** - IL8, **F**, **H** Claudin 4, and **I** NRF1). #p < 0.05, ##p < 0.01, ###p < 0.001 vs. CTRL; *p < 0.05, **p < 0.01, ***p < 0.001 vs. HFD.

To determine whether EAA supplementation protected intestinal barrier structure in obesity, we assessed gut morphometric parameters (weight and length), inflammation, and TJ integrity (Rohr et al., 2020). HFD-fed, mice exhibited reduced intestinal length and weight—hallmarks of intestinal dysfunction—accompanied by increased plasma calprotectin, an inflammation marker (Malham et al., 2019), and higher expression of pro-inflammatory cytokines (IL6, IL8, and TNFα) in jejunal tissue, all of which were attenuated or reversed by EAAs (Figures 3A–C). Accordingly, plasma levels of cystine, reduced in HFD-EAA-fed mice (therapeutic schedule), was positively correlated with intestinal weight, suggesting a potential link with gut structural changes (Supplementary Table S2). Plasma citrulline—a non-proteinogenic amino acid predominantly synthesized by small intestinal enterocytes and recognized as a biomarker of enterocyte mass and barrier function (Crenn et al., 2008)—was markedly reduced in HFD-fed mice and restored by EAAs (Figure 3D). Notably, citrulline positively correlated with branched-chain amino acids (BCAAs), linking EAA status to gut epithelial health (Supplementary Table S2). At the structural level, HFD induced TJ remodeling with downregulation of ZO-1, occluding, E-cadherin, and barrier-forming claudins (1, 3, 4, 7, 15), alongside upregulation of pore-forming claudin-2 (Figures 3E–H) (Ahmad et al., 2017). EAA supplementation preserved TJ and AJ expression and prevented claudin 2 induction, indicating intact epithelial integrity. Given the central role of mitochondria in epithelial renewal and barrier maintenance, we assessed mitochondrial markers. HFD markedly reduced jejunal expression of PGC-1α and other genes involved in mitochondrial biogenesis, consistent with mitochondrial dysfunction (Guerbette et al., 2022; 2025) (Figure 3J). EAA supplementation restored their expression, supporting the preservation of mitochondrial function (Figure 3J). These findings show that EAAs counteract HFD-induced intestinal inflammation and barrier dysfunction by modulating the expression of genes involved in mitochondrial biogenesis and epithelial integrity, suggesting a potential mechanism for reducing the ‘leaky gut’ phenotype typical of obesity.

### 3.4 E7 improves intestinal barrier integrity by stimulating mitochondrial biogenesis *in vitro*

**FIGURE 4:**
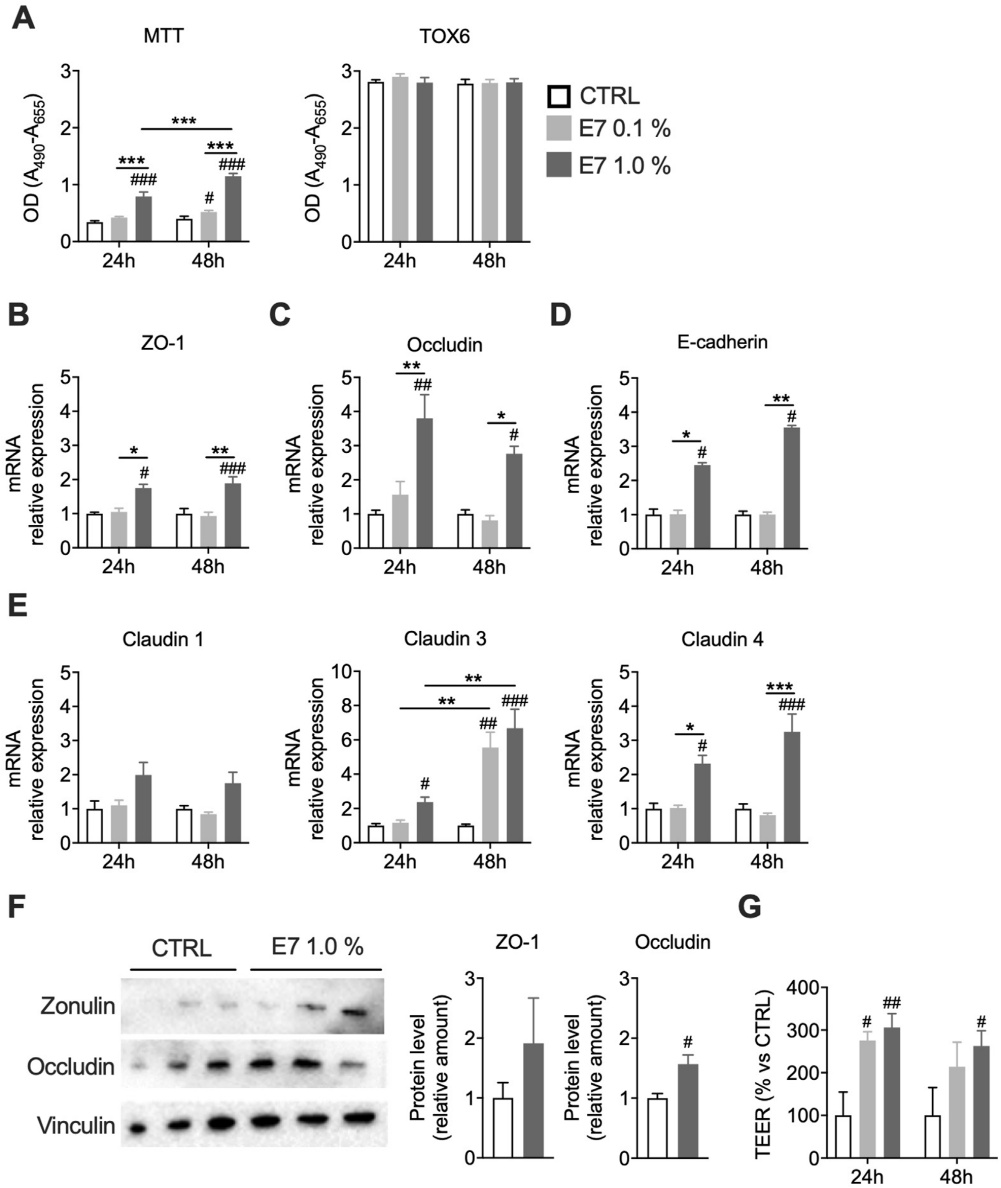
E7 improves intestinal barrier function in an *in vitro* Caco-2 model. Caco-2 cells differentiated for 14 days were treated with E7 (0.1 or 1.0%) for 24 h or 48 h. **(A)** Cell viability assessed by MTT and TOX6 assays. **(B–E)** mRNA levels of intestinal permeability markers. Transcript levels were normalized to GAPDH and expressed relative to untreated control (CTRL) cells (set as 1.0). **(F)** Western blot analysis of ZO-1 and occluding protein levels in cells treated for 48 h. Data are presented as relative amounts normalized to CTRL (set as 1.0); representative blots from three independent experiments are shown. **(G)** Transepithelial electrical resistance (TEER) across the monolayer, expressed as percentage relative to CTRL. Data are mean ± SEM (**A–E**: n = 4/group; **F, G** n = 3/group). Two-way ANOVA **A–E and G**) or unpaired Student’s t-test **(F)**. #p < 0.05, ##p < 0.01, ###p < 0.001 vs. CTRL.

To dissect whether EAAs act directly on IECs, we used differentiated Caco-2 cell monolayers as an *in vitro* intestinal barrier model (Fogh J and Trempe G, 1975; Engle et al., 1998; Haddad et al., 2023). To define the optimal experimental conditions, we performed dose- and time-response studies. Differentiated Caco-2 monolayers (14 and 21 days post-confluence) were treated with E7 for either 24 or 48 h. E7 treatment (0.1%–1.0%) did not reduce cell viability. Instead, the MTT assay indicated increased cell viability not detected by the TOX6 test (Figure 4A), suggesting enhanced mitochondrial activity, as MTT primarily reflects dehydrogenase activity, including mitochondrial succinate dehydrogenase (Rai et al., 2018). Under basal conditions, E7 upregulated TJ and AJ markers (ZO-1, occludin, claudin 1/3/4, E-cadherin), most effectively in differentiated cells (14 days) treated with 1% E7 for 48 h (Figures 4B–F; Supplementary Figures S4A–D). This was accompanied by increased TEER, indicating improved barrier function (Figure 4G).

**FIGURE 5:**
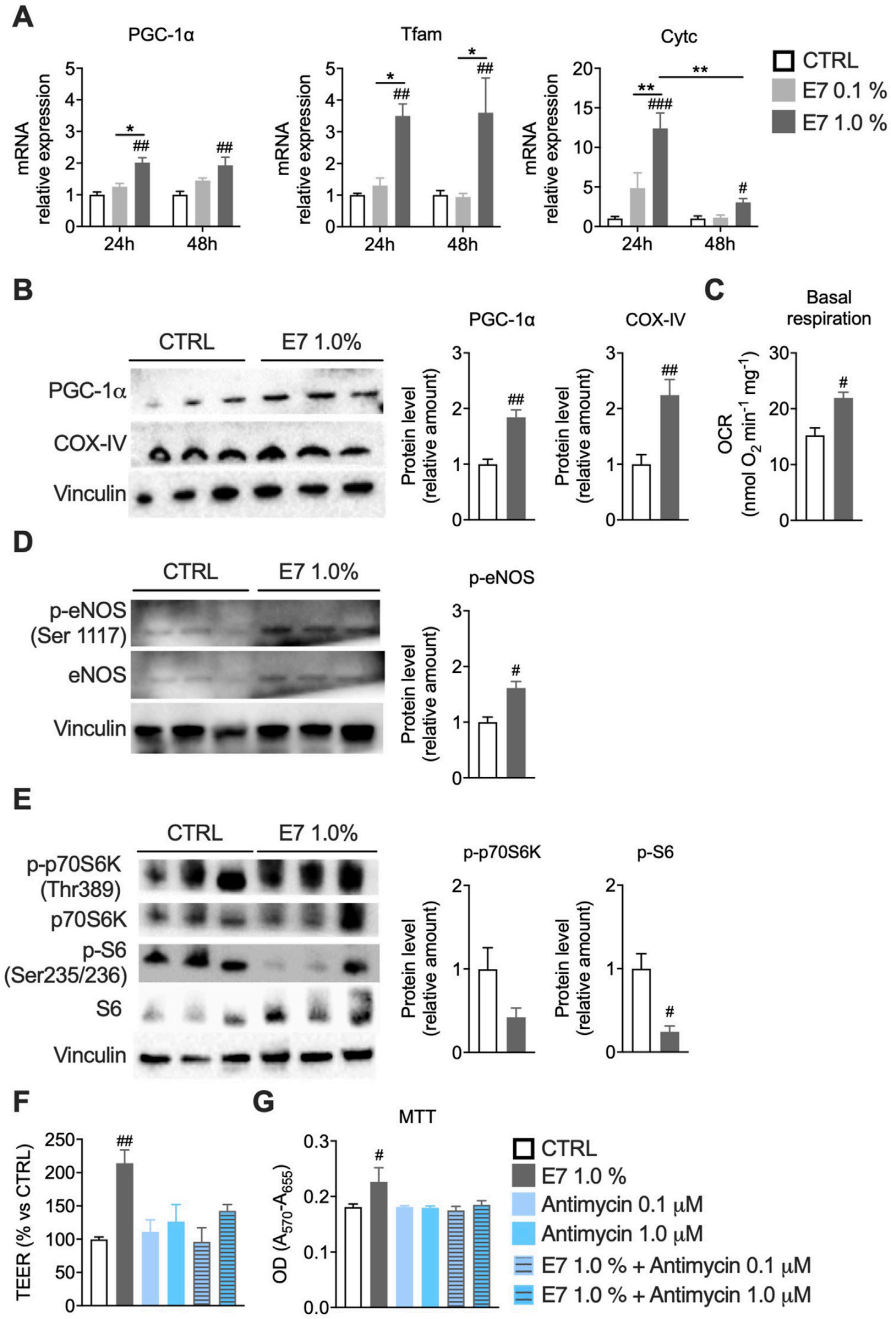
E7 enhances intestinal barrier function by stimulating mitochondrial activity. **(A)** mRNA levels of mitochondrial biogenesis genes. Transcript levels were normalized to GAPDH and expressed relative to untreated control (CTRL) cells (set as 1.0). **(B)** Western blot analysis of PGC-1α and COX-IV protein levels in Caco-2 cells treated for 48 h. Data are presented as relative to CTRL (set as 1.0); representative blots from three independent experiments are shown. **(C)** Basal OCR measured with a Clark’s electrode in Caco-2 cells treated for 48 h; OCR normalized to total protein content. **(D)** Western blot analysis of phosphorylated-eNOS (p-eNOS) normalized to total eNOS. **(E)** Western blot analysis of phosphorylated p70S6K normalized to total p70S6K and phosphorylated S6 normalized to total S6. **(F)** TEER in Caco-2 monolayers (14 days) pre-treated with E7 (1.0%) for 24 h and then exposed to antimycin A (0.1–1.0 µM) for other 24 h; TEER expressed as % vs. CTRL. **(G)** Cell viability (MTT) in differentiated Caco-2 cells pre-treated with E7 (1.0%) and exposed to antimycin A (0.1–1.0 µM) for 24 h. Data are mean ± SEM (**A**: n = 4/group; **B–F**: n = 3/group; **G** n = 5/group). Two-way ANOVA **(A, F, G)**, unpaired Student’s t-test **(B–E)**, Mann-Whitney test (B–right). #p < 0.05, ##p < 0.01, ###p < 0.001 vs. CTRL.

E7 enhanced mitochondrial biogenesis mainly in differentiated Caco-2 cells (14 days), as shown by upregulation of PGC-1α, Tfam, Cytc, and COX-IV, and increased OCR (+44% vs. control) (Figures 5A,B; Supplementary Figure S4E). These effects were linked to elevated eNOS phosphorylation, consistent with known EAA-induced mitochondrial activation (Figure 5D) (Nisoli et al., 2008). Interestingly, E7 reduced phosphorylation of mTORC1 effectors, significantly decreasing S6 phosphorylation and showing a trend toward reduced p70S6K phosphorylation (Figure 5E). These data suggest that the observed barrier improvement and mitochondrial stimulation involve mTORC1 inhibition, a mechanism associated with improved gut permeability under chronic stress, in accordance with Kaur et al. (Kaur and Moreau, 2019; 2021).

To determine whether E7’s barrier-protective action relies on mitochondrial function, we challenged Caco-2 monolayers with antimycin A, a complex III inhibitor. While antimycin A alone did not significantly alter TEER under our conditions, pre-treatment with E7 (1.0%) failed to enhance TEER in the presence of antimycin A (0.1–1.0 µM), indicating a loss of E7’s barrier benefit during mitochondrial inhibition (Figure 5F). Importantly, cell viability remained unchanged across conditions (Figure 5G). These data support that E7 improves barrier integrity through mitochondrial activation rather than non-specific trophic effects.

In addition, we confirmed that the inclusion of Krebs cycle intermediates in E7 resulted in greater efficiency than the standard EAA mixture (*i.e.*, without Krebs cycle intermediates) used *in vivo* (Supplementary Figure S5) (Brunetti et al., 2020; Tedesco et al., 2020b). For this reason, we chose to use E7 for the *in vitro* experiments.

In summary, these findings indicate that E7 exerts a direct effect on enterocytes by promoting mitochondrial biogenesis, closely associated with improved intestinal barrier function. Preliminary data identified 14 days of differentiation as the optimal time point for evaluating the effects of E7, providing the basis for subsequent experiments under inflammatory conditions. Additionally, treatment with E7 at 1.0% proved most effective at both 24 and 48 h, consistent with our previous studies in other cell types (Tedesco et al., 2018; 2020a; 2022; Ruocco et al., 2020). Notably, the E7 concentrations used *in vitro* were lower than the peak luminal EAA levels estimated *in vivo* (Supplementary Figure S2D), yet still elicited significant protective effects. This observation strengthens the translational value of our *in vitro* model, indicating that even lower luminal concentrations of EAAs are sufficient to promote mitochondrial function and barrier integrity in IECs.

### 3.5 E7 preserves intestinal barrier integrity and reduces inflammation *in vitro*

We next investigated whether E7 protects IECs under inflammatory stress. Differentiated Caco-2 monolayers, a well-established model of gut epithelium, respond to IS by producing cytokines such as IL8 (Hollebeeck et al., 2012; Rodríguez-Ramiro et al., 2013; Ponce de León-Rodríguez et al., 2019). Cells were exposed to IL-1β, TNF-α, and LPS (*i.e.*, IS) for 24–72 h, with or without E7 pre-treatment (1.0%). Since cytokine release typically represents an early response (12–24 h), whereas epithelial barrier disruption—manifested by reduced TEER and TJ protein expression—requires prolonged exposure (≥48 h) (Al-Sadi et al., 2014), we evaluated effects at 24, 48, and 72 h. As previously observed, neither E7 nor IS compromised cell viability (Supplementary Figure S6).

**FIGURE 6:**
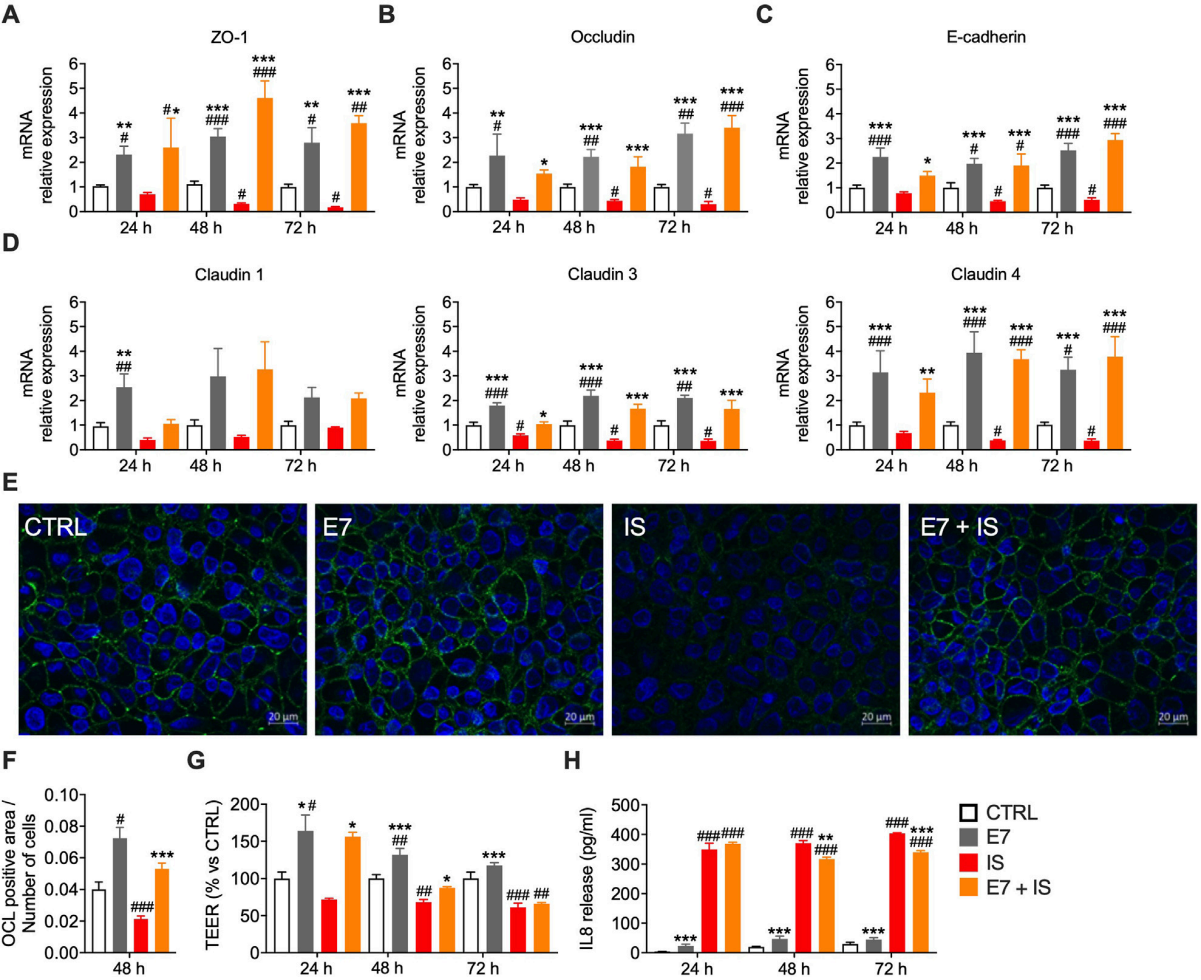
E7 preserves intestinal barrier integrity and exerts anti-inflammatory effects in an *in vitro* model of gut inflammation. **(A–D)** mRNA levels of intestinal permeability markers in Caco-2 cells differentiated for 14 days and treated with E7 (1.0%) ± inflammatory stimuli (IS) for 24 h, 48 h, or 72 h. Transcript levels were normalized to GAPDH and expressed relative to untreated control (CTRL) cells (set as 1.0). **(E,F)** Immunofluorescence analysis of occluding in Caco-2 cells differentiated for 14 days and treated with E7 (1.0%) ± IS for 48 h. Representative images of occludin staining (green); cell nuclei were stained with DAPI; scale bar = 20 µm **(E)**. Quantification of occludin-positive area (n = 25 fields) **(F)**. **(G)** TEER in Caco-2 monolayers (14 days post-differentiation) pre-treated with E7 (1.0%) for 24 h, then exposed to IS for 24–72 h. TEER is expressed as percentage relative to CTRL. **(H)** IL8 secretion in culture medium from Caco-2 cells pre-treated with E7 (1.0%) for 1 h, then exposed to IS for 24–72 h; values expressed in pg/mL. Data are mean ± SEM (**A–D**: n = 6/group; **F**: n = 3/group; **G**: n = 5/group). Two-way ANOVA **(A–D, G, H)** or one-way ANOVA **(F)**. #p < 0.05, ##p < 0.01, ###p < 0.001 vs. CTRL; *p < 0.05, **p < 0.01, ***p < 0.001 vs. IS.

Under basal conditions, E7 enhanced mRNA and protein levels of TJ and AJ components (ZO-1, occludin, E-cadherin, claudins), with effects sustained up to 72 h (Figures 6A–F), confirming our prior findings. Exposure to IS markedly disrupted barrier integrity, reducing both gene and protein expression, whereas co-treatment with E7 prevented these alterations, preserving TJ architecture (Figures 6A–F). TEER measurements confirmed functional protection. E7 increased TEER under basal conditions, although this effect declined over time (Figure 6G). IS reduced TEER beginning at 48 h (−30% vs. CTRL at 72 h), but E7 significantly mitigated this decline, improving TEER by +26% vs. IS at 24 h and preserving barrier integrity at 48 h (−17% vs. CTRL; +12% vs. IS) (Figure 6G). At 72 h, however, protection diminished, likely reflecting amino acid depletion (Figure 6G).

To assess anti-inflammatory effects, IL8 secretion was quantified. As expected, IS induced a robust IL8 response (+99-fold vs. CTRL at 24 h), persisting through 72 h (Figure 6H). E7 reduced IL8 release at 48 h (−15% vs. IS), and 72 h (−16% vs. IS), even when TEER protection was waned (Figures 6G,H). Together, these findings demonstrate that E7 preserves barrier integrity and function while attenuating cytokine secretion under inflammatory stress, with efficacy sustained for up to 48 h.

### 3.6 E7 prevents mitochondrial dysfunction and metabolic shift during inflammation *in vitro*

Mitochondria are central to epithelial energy metabolism, survival, and immune regulation (Rath et al., 2018). Mitochondrial dysfunction is common in gastrointestinal diseases characterized by barrier disruption, including obesity-associated gut inflammation (Wang et al., 2014; Guerbette et al., 2024). It involves downregulation of electron transport chain (ETC.) genes, impaired oxidative phosphorylation, reduced respiration, altered membrane potential, and excessive ROS production (Chernyavskij et al., 2023), all of which compromise TJ integrity and increase epithelial permeability. While basal mitochondrial ROS support epithelial renewal, excessive ROS coupled with diminished antioxidant defenses (*e.g.*, catalase, SOD2) drives oxidative damage and apoptosis (Chernyavskij et al., 2023).

**FIGURE 7:**
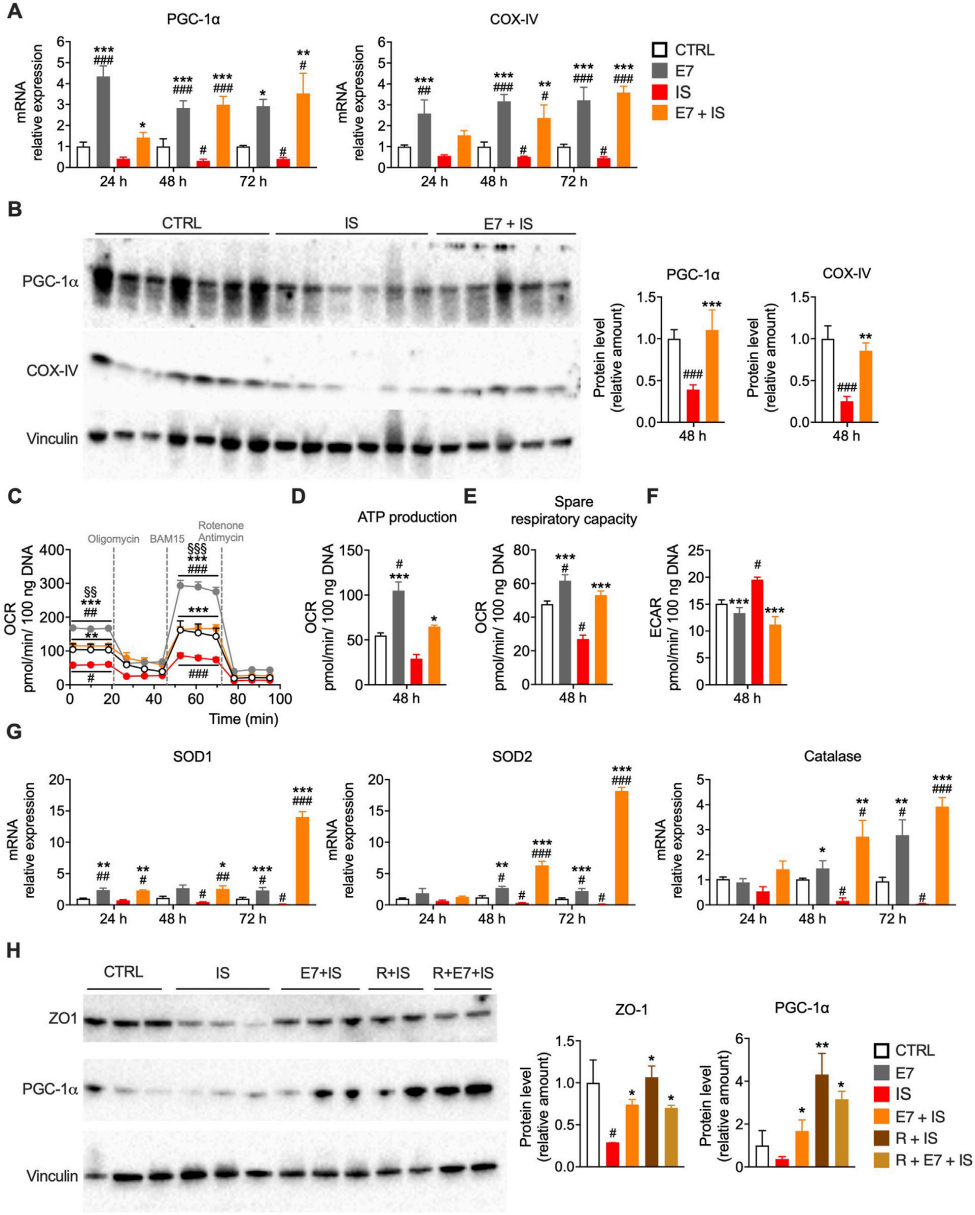
E7 prevents mitochondrial dysfunction and metabolic shift during inflammation in Caco-2 cells. **(A)** mRNA levels of mitochondrial genes in Caco-2 cells differentiated for 14 days and treated with E7 (1.0%) ± IS for 24, 48, or 72 h. **(B)** Western blot analysis of mitochondrial protein markers in cells treated for 48 h. Data are presented as relative amounts normalized to CTRL (set as 1.0); representative blots from three independent experiments. **(C–F)** Mitochondrial respiration analysis using Seahorse XFe24 in Caco-2 cells treated for 48 h: oxygen consumption rate (OCR) **(C)**, ATP production **(D)**, spare respiratory capacity (maximal respiration minus basal respiration) **(E)**, and extracellular acidification rate (ECAR) as a measure of glycolysis **(F)**. **(G)** mRNA levels of antioxidant enzyme genes, normalized to GAPDH and expressed relative to CTRL (set as 1.0). **(H)** Western blot analysis of ZO-1 (gut permeability marker) and PGC-1α (mitochondrial biogenesis marker) in cells treated for 48 h with E7 ± IS, with or without rapamycin (R, 50 nM). CTRL values were set as 1.0. Data are as mean ± SEM (**A, G**: n = 6/group; **B**: n = 6–7/group; **C–F**: n = 5/group; **H**: n = 2–3/group). Two-way ANOVA **(A,C and G)**, one-way ANOVA **(B,E,F and H)** or or Kruskal–Wallis test **(D)**. #p < 0.05, ##p < 0.01, ###p < 0.001 vs. CTRL; *p < 0.05, **p < 0.01, ***p < 0.001 vs. IS.

To determine whether E7 mitigates inflammation-induced mitochondrial dysfunction, we assessed mitochondrial biogenesis and respiration in Caco-2 cells exposed to IS ± E7. IS significantly reduced PGC-1α and COX-IV expression (mRNA and protein) at 48 h, whereas E7 prevented this decline at all time points (Figures 7A,B). Mitochondrial function, assessed *via* OCR, was markedly impaired by IS, with reductions in basal (∼43%), ATP-linked respiration (∼47%) and spare respiratory capacity (∼44%) (Figures 7C–E), consistent with prior reports of cytokine-induced mitochondrial dysfunction (Crakes et al., 2019). IS also induced a metabolic shift toward glycolysis, evidenced by increased ECAR values (Figure 7F), similar to findings in endothelial cells (Xiao et al., 2021). E7 restored oxidative phosphorylation and attenuated this glycolytic shift, promoting a more energy-efficient phenotype. Inflammation also downregulated antioxidant enzymes, suggesting excessive ROS involvement (Figure 7G). E7 prevented this downregulation and enhanced antioxidant enzyme expression, indicating an antioxidant effect that may contribute to its anti-inflammatory action (Figure 7G).

To assess the role of mTORC1, we treated differentiated Caco-2 cells with rapamycin (an mTORC1 inhibitor, 50 nM) ± E7 (1.0%, 48 h) under IS. Both rapamycin and E7 individually preserved ZO-1 and PGC-1α expression, while their combination showed no additive effect (Figure 7H), suggesting that E7 may act partly through mTORC1 inhibition. This result is consistent with previous studies in which Caco-2 cells treated with rapamycin exhibited improved gut permeability in association with mTORC1 inhibition (Xu et al., 2024). Together, these findings link inflammation-induced gut barrier disruption to mitochondrial dysfunction. E7 counteracts the IEC damage by preserving mitochondrial biogenesis, restoring respiration, reducing glycolytic shift, and enhancing antioxidant defenses. Its overlap with rapamycin implicates mTORC1 modulation as part of its mechanism. Thus, E7 exerts anti-inflammatory effects through integrated mitochondrial and signaling pathways that sustain epithelial barrier integrity.

## 4 Discussion

Unhealthy diets compromise the gut barrier, driving chronic inflammation that contributes to IBDs, obesity, diabetes, autoimmune, and aging-related diseases (Martel et al., 2022). High-fat food intake, in particular, disrupts barrier function by inducing mucosal inflammation and dysbiosis, which contribute to systemic endotoxemia and metabolic complications (Genser et al., 2018; Mouries et al., 2019). Mitochondria in IECs are crucial for maintaining barrier homeostasis (Rath et al., 2018). However, chronic inflammation and HFD impair mitochondrial function, weaken mucosal integrity, and disrupt key proteins regulating permeability (Guerbette et al., 2022; 2024; 2025).

Lifestyle modification remains one of the most effective strategies to prevent and reverse obesity and its complications. A critical question is whether dietary interventions can improve conditions associated with increased gut permeability. Evidence from both human and animal studies suggests that several approaches, including dietary fiber reintroduction (Krawczyk et al., 2018), caloric restriction (Ott et al., 2017), intermittent fasting and fasting-mimicking diets (Rangan et al., 2019; Liu et al., 2020) and supplementation with bioactive compounds such as berberine (Amasheh et al., 2010), curcumin (Feng et al., 2019), quercetin (Suzuki and Hara, 2009), and resveratrol (Mayangsari and Suzuki, 2018), enhance barrier integrity and attenuate inflammation. However, despite its robust health benefits in preclinical models, caloric restriction and intermittent fasting are notably difficult to sustain in humans over time, and frequently result in weight regain, with high heterogeneity across participants (Langeveld and DeVries, 2015; Wang et al., 2025). In parallel, most bioactive compounds are characterized by poor bioavailability and rapid metabolism, which significantly limits their translational potential and often necessitates advanced formulations to achieve clinically meaningful efficacy (Smith et al., 2011; Pannu and Bhatnagar, 2019; Ai et al., 2021; Hegde et al., 2023).

Here, we propose a nutritional approach based on the administration of EAAs. Dietary EAA supplementation has been extensively shown to regulate metabolism and energy balance by directly modulating peripheral tissues such as muscle, adipose tissue, heart and liver. EAAs promote mitochondrial biogenesis (Nisoli et al., 2008; D’Antona et al., 2010; Valerio et al., 2011; Ragni et al., 2023), protect against oxidative damage (Corsetti et al., 2014; D’Antona et al., 2016; Tedesco et al., 2020a), enhance protein synthesis and physical endurance (Yamamoto et al., 2010; Nisoli et al., 2015), reduce body weight and improve glucose and lipid metabolism (Cota et al., 2006; Binder et al., 2013; 2014; Ruocco et al., 2020), stimulate energy expenditure (Ruocco et al., 2020; 2023), and strengthen immune function (Bassit et al., 2002; Aquilani et al., 2011). Collectively, these effects contribute to improved metabolic health and lifespan (Ruocco et al., 2021). Notably, the action of EAAs is context-dependent, in catabolic states they serve primarily as energy substrates, whereas in anabolic conditions they fuel protein synthesis and cell growth (Bifari and Nisoli, 2017). Clinical data further highlight the therapeutic potential of amino acid supplementation. EAAs enhances physical and cognitive performance in the elderly, improving mitochondrial biogenesis in peripheral blood mononuclear cells (Buondonno et al., 2019), preserves muscle mass during weight loss (Brunani et al., 2023), reduces infection risk (Aquilani et al., 2011), and is safe with no reported side effects. Furthermore, amino acids play a critical role in gut homeostasis, regulating structural integrity, epithelial turnover, redox balance, immune responses, and microbial composition. Inflammatory conditions, such as LPS challenge, disrupt amino acid metabolism and reduce digestibility, whereas supplementation with tryptophan, phenylalanine, or tyrosine improves amino acid sensing and exerts anti-inflammatory effects (Duanmu et al., 2022). Glutamine has also been shown to reduce gut permeability, endotoxemia, and inflammation in postoperative patients and to improve barrier function in malnourished children (Quan, 2004; Lima et al., 2005). In elderly patients with chronic kidney disease, EAAs reduced intestinal inflammation and improved barrier function (Aquilani et al., 2022).

Based on these concepts, our study extends the focus to EAAs, which—unlike single amino acids—simultaneously target both epithelial bioenergetics and nutrient-sensing pathways. We hypothesized that EAAs could be particularly beneficial in the context of intestinal damage, where mitochondrial dysfunction in IECs is well documented (Guerbette et al., 2022). Our results demonstrate that dietary EAAs provide substantial protection against obesity-induced intestinal barrier dysfunction by preserving mitochondrial function in IECs. Using a combined *in vivo* and *in vitro* approach and building on our previous work (Ruocco et al., 2020), we show that EAAs both prevent and reverse HFD-induced barrier impairment.

We show that dietary supplementation with EAAs prevents and reverses obesity and metabolic dysfunction in DIO mice by restoring intestinal barrier integrity, reducing inflammation, and sustaining mitochondrial biogenesis. EAA-fed mice exhibited reduced visceral fat, particularly in mesenteric depots, which likely contributes to attenuated systemic and intestinal inflammation, in line with previous evidence linking adiposity to gut barrier dysfunction and IBD pathogenesis (Kredel and Siegmund, 2014; Ha et al., 2020). EAAs also modulated nutrient absorption and gut metabolism. In HFD-fed mice, reduced fecal output was associated with increased caloric absorption, suggesting non-selective nutrient uptake driven by impaired barrier function (Murphy et al., 2015). In contrast, EAA-fed mice displayed enhanced protein and amino acid absorption, evidenced by decreased fecal nitrogen, upregulation of LAT1 and mitochondrial SLC25A44, and elevated plasma amino acid levels. These findings support a model in which EAAs promote selective nutrient uptake, likely preserving mitochondrial activity in intestinal epithelial cells and supporting intestinal homeostasis.

Correlation analyses further underscore the role of EAAs in nutrient sensing and metabolic regulation. Asparagine, phenylalanine, and tyrosine, elevated in HFD mice and normalized by EAA treatment, were positively associated with impaired glucose homeostasis and increased eWAT mass, while arginine, histidine, lysine, and tryptophan correlated with fecal output, suggesting differences in absorption efficiency. By promoting amino acid uptake while limiting non-specific absorption of carbohydrates and lipids, EAAs may reduce net caloric intake while preserving mitochondrial function and energy expenditure, thereby enhancing metabolic efficiency and limiting fat accumulation. This interpretation agrees with our previous findings showing that EAAs stimulate energy expenditure through BAT thermogenesis, contributing to weight loss and improved glycemic control (Ruocco et al., 2020). Nonetheless, part of the beneficial metabolic effect may also derive from reduced net caloric intake. Importantly, EAAs protected against HFD-induced intestinal barrier remodeling. HFD feeding reduced intestinal length and weight, increased pro-inflammatory cytokines (IL6, IL8, TNFα), altered TJ and AJ expression, lowered enterocyte-derived citrulline (a marker of enterocyte mass and barrier integrity) (Crenn et al., 2008), and elevated calprotectin (a marker of inflammation) (Malham et al., 2019), collectively indicating barrier damage and chronic inflammation. This aligns with Cani et al., who demonstrated that HFD-induced elevations in circulating LPS trigger metabolic disturbances through TJ disruption and microbial translocation (Cani et al., 2007). EAA supplementation prevented and reversed these alterations by restoring TJ and AJ, normalizing barrier-forming claudins, and suppressing pore-forming claudin-2. These effects were accompanied by preserved mitochondrial biogenesis markers and electron transport chain gene expression, reinforcing the link between gut barrier integrity and mitochondrial function (Rath et al., 2018; Crakes et al., 2019). Furthermore, increased eNOS gene expression suggests sustained mitochondrial biogenesis (Nisoli, 2003; Nisoli et al., 2005; 2008). This is consistent with our previous work demonstrating that replacing dietary protein with an EAA mixture can both prevent and reverse obesity and glucose intolerance. In mice fed a low-fat diet, EAA supplementation further improved gut homeostasis by enhancing microbiota composition, accelerating intestinal transit, promoting villus elongation, and maintaining fecal energy efficiency despite a reduction in excreted mass (Ruocco et al., 2020).

*In vitro*, E7 directly preserves epithelial barrier integrity and exerts anti-inflammatory effects. However, its direct action on IECs and the differences with *in vivo* treatment require careful interpretation. *In vivo*, the intestinal epithelium is exposed to EAAs only transiently. Plasma EAA levels rise within 30–150 min after ingestion, with luminal concentrations peaking immediately after feeding and then rapidly declining due to absorption, bacterial metabolism, and intestinal transit (Rondanelli et al., 2017). Free amino acid concentrations in the intestinal lumen are typically 50–300 µM after a standard meal, but can increase to 0.6–6 mM following a protein-rich meal (Bröer, 2023). Under our experimental conditions, we estimated peak luminal concentrations at ∼ 6 M immediately post-ingestion. By contrast, *in vitro*, Caco-2 cells were continuously exposed to E7 (1.0%; 66 mM) for 24–72 h. In this setting, amino acids were gradually metabolized but remained available for an extended period. Thus, although the initial concentrations *in vitro* were nearly 100-fold lower than peak luminal levels *in vivo*, sustained exposure was sufficient to elicit robust protective effects. This highlights the translational value of our model, continuous exposure of Caco-2 cells to sub-luminal EAA concentrations avoids transient peaks and enables a more realistic assessment of the long-term benefits of amino acids on epithelial barrier function.

Mechanistically, E7 promoted mitochondrial biogenesis, enhanced eNOS phosphorylation and respiratory function, and prevented inflammation-induced metabolic reprogramming toward glycolysis (Xiao et al., 2021). In addition, E7 upregulated antioxidant enzymes such as SOD2 and catalase, thereby reducing ROS-mediated epithelial injury (Chernyavskij et al., 2023). These results are consistent with and further support findings obtained *in vivo*. Our results also implicate mTORC1 as a mediator of these effects. Leucine-driven mTORC1 activation is known to support enterocyte proliferation and nutrient transport in intestinal porcine enterocytes, particularly following acute exposure of up to 10 h (Zhang et al., 2014). However, while transient activation of mTORC1 promotes epithelial renewal and barrier integrity, chronic activation has been linked to exacerbated intestinal inflammation, whereas inhibition under pathological conditions can restore barrier function (Kaur and Moreau, 2019; 2021; Kotani et al., 2020). In our study, E7 reduced phosphorylation of p70S6K and S6, consistent with mTORC1 inhibition. Moreover, rapamycin reproduced the protective effects of E7 on ZO-1 and PGC-1α in inflamed cells, as also reported by Xu et al. in ulcerative colitis (Xu et al., 2024). No additive effect was observed with combined treatment, supporting mTORC1 may be a shared target. Together, these findings suggest that E7 mediates barrier protection directly in IECs, stimulating mitochondrial biogenesis *via* the eNOS/PGC-1α axis and reducing mTORC1 activity under inflammatory stress. In addition, our data indicate a catabolic state in intestinal epithelial cells, in which EAAs may be preferentially utilized as energy substrates. This metabolic shift further supports their role in sustaining epithelial homeostasis under stress conditions. Our *in vitro* studies showed further that the inclusion of Krebs cycle intermediates in the E7 formulation enhances mitochondrial biogenesis and supports epithelial homeostasis more effectively than the standard EAA mixture without intermediates, suggesting a synergic interaction between amino acids and citrate succinate and malate. This is consistent with our previous work, in which E7 was more effective than the EAA mixture in promoting mitochondrial biogenesis in cardiomyocytes (Tedesco et al., 2020b). Conceptually, these intermediates can fuel anaplerosis, sustain NADH/FADH_2_ generation, and improve redox balance, thereby reinforcing oxidative phosphorylation and junctional homeostasis. Consistent with this rationale, succinate can counteract HFD-induced barrier dysfunction by influencing epithelial permeability, immune signalling, goblet cell differentiation, and microbiota composition (Li et al., 2019; 2023). Citric acid has been shown to strengthen TJs and improve mucosal immunity in porcine models of enterotoxaemia (Liu et al., 2021; Hu et al., 2024), and malic acid supplementation reduced oxidative stress and inflammatory signatures *via* microbiota–metabolite modulation (Chen et al., 2024). Mechanistically, in addition to their metabolic role, succinate–SUCNR1 (succinate receptor 1) signalling may contribute to epithelial and immune crosstalk, while improved mitochondrial flux can secondarily modulate mTORC1 and promote the eNOS–PGC-1α program we observed with E7. However, while supportive studies exist across species and models (Li et al., 2019; 2023; Liu et al., 2021; Chen et al., 2024; Hu et al., 2024), direct evidence in human HFD-associated gut leak is limited and represents a priority for translational validation.

We acknowledge several limitations in our work. Although we demonstrated e superior effect of E7 compared with EAA mixture *in vitro*, the specific contribution of each intermediate remains to be quantified. Future work will include add-back/subtraction experiments, pharmacologic receptor modulation (*e.g.*, SUCNR1 antagonism), and *in vivo* comparisons under HFD feeding. Moreover, while our data strongly suggest that preservation of mitochondrial function is central to maintaining gut barrier integrity and preventing inflammatory damage, a direct causal demonstration is still lacking and will require further investigation. Furthermore, although we observed direct effects of EAAs on IECs, potential interactions with other intestinal cell type (*e.g.*, goblet cell), gut microbiota and the immune system cannot be excluded. Amino acids can be utilized by enterocytes or metabolized by intestinal bacteria generating NO and short-chain fatty acids (acetate, propionate, butyrate) which influence epithelial barrier function and immune responses (Bifari et al., 2017). Additionally, microbial metabolites can modulate mitochondrial activity and mitochondrial function shapes the microbial niche. Disruption of this bidirectional signaling contributes to gut inflammation (Jackson and Theiss, 2020).

Amino acids are key modulators of immune function. Dietary amino acid restriction (*i.e.*, during malnutrition) impairs cytotoxic T lymphocyte and natural killer cell activity and contributes to immune-senescence (Lesourd, 2009). Conversely, EAA supplementation preserves immune competence in several pathological conditions (*e.g.*, cirrhosis, surgical recovery, rehabilitative), *via* effects on lymphocyte and macrophage activation and cytokine production (Nuwer et al., 1983; Tsukishiro et al., 2000; Kakazu et al., 2009; Aquilani et al., 2011; 2021; Boselli et al., 2012). Among individual amino acids, phenylalanine can influence immunity directly, by regulating NO synthesis in leukocytes, and indirectly through conversion to tyrosine, which supports catecholamine synthesis and immune-cell signalling (Shi et al., 2004). Likewise, leucine and other BCAAs modulate immune activity by tuning mTORC1 signalling in T cells (Powell and Delgoffe, 2010; Sinclair et al., 2013). Specific amino acids also contribute to intestinal integrity by shaping gut-immune responses (Ruth and Field, 2013). For example, tryptophan metabolism by commensal microbiota generates indole derivatives that activate the aryl hydrocarbon receptor, promoting IL22 production by group 3 innate lymphoid cells and Th17 cells, enhancing antimicrobial defence and epithelial repair (Qiu et al., 2012; Zelante et al., 2013). In addition, threonine serves as an essential substrate for goblet-cell mucin biosynthesis, thereby supporting the mucus barrier and limiting paracellular permeability (Faure et al., 2005). These mechanisms align with emerging evidence that Th17–IL22 signalling influences epithelial lipid absorption and barrier integrity in response to HFD feeding (Gao et al., 2025). We further hypothesize that increased NO bioavailability and mTORC1 modulation converge with enhanced mitochondrial activity in immune cells, consistent with our previous findings that the EAA mixture containing phenylalanine and BCAAs stimulated mitochondrial biogenesis in PBMCs (Buondonno et al., 2019). Finally, growing evidence indicates that Krebs cycle intermediates, such as citrate and succinate, modulate macrophage activation, cytokine production, and gut epithelial barrier function (Li et al., 2019; 2023; Liu et al., 2021; Hu et al., 2024), suggesting potential synergy with EAAs in sustaining gut and immune homeostasis. Although we did not assess mucosal immune cells here, this framework provides a biologically plausible immune–epithelial crosstalk that could contribute to the benefits observed with EAA/E7. Accordingly, future studies should comprehensively investigate the direct effects of EAAs and Krebs cycle intermediates on microbiota dynamics and immune regulation, and determine whether activation of these pathways complements the epithelial, mitochondria-dependent effects observed in the present study.

Taken together, our study identifies EAAs as a promising nutritional strategy to preserve intestinal barrier integrity and counteract obesity-related gut dysfunction. EAAs support mitochondrial biogenesis, modulate mTORC1 signaling, and reduce epithelial inflammation, providing a mechanistic basis for restoring gut barrier function in metabolic and inflammatory disorders. While preclinical and some clinical data are encouraging, validation in human obesity cohorts is needed. Overall, EAAs offer a strategy to target interconnected mechanisms—mitochondrial dysfunction, barrier disruption, and systemic metabolic complications—within integrated lifestyle and therapeutic approaches.

## Data Availability

The raw data supporting the conclusions of this article will be made available by the authors, without undue reservation.
