# Supplementary material for: Essential amino acids preserve intestinal barrier integrity via mitochondrial protection in obesity and gut inflammation
Source: Front Pharmacol. 2025 Dec 3;16:1694723. doi: 10.3389/fphar.2025.1694723 (PMC12708562; doi:10.3389/fphar.2025.1694723)
Supplement: Supplementary file 1 [file DataSheet1.docx]

# Supplementary Figures and Tables


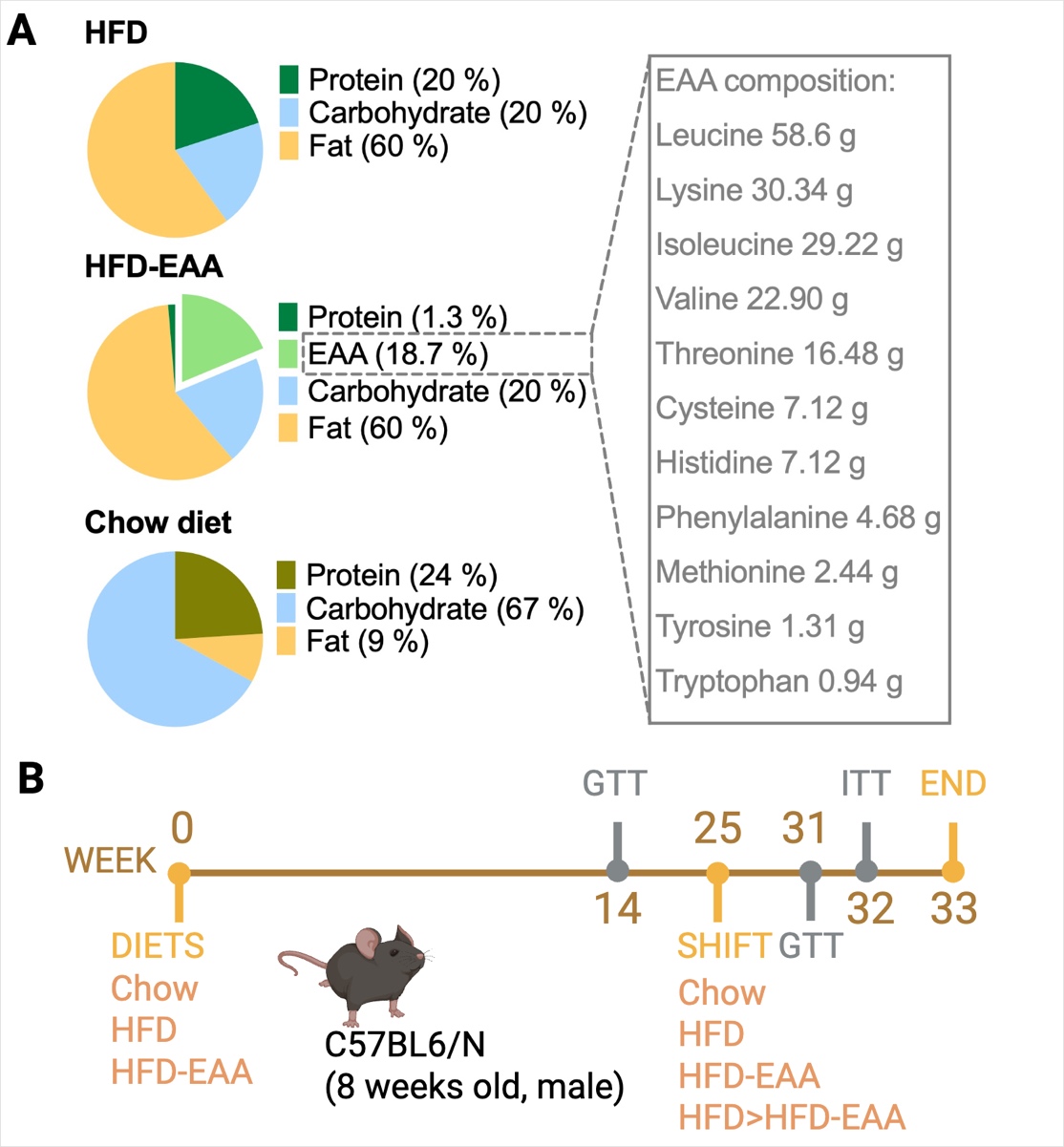


**Supplementary Figure 1. Diet composition and *in vivo* experimental design.** (A) Macronutrient composition of the diets and essential amino acid (EAA) profile of the experimental diets, expressed as g per 200 g of protein per 1,000 g diet. (B) *In vivo* treatment schedule (created with BioRender).


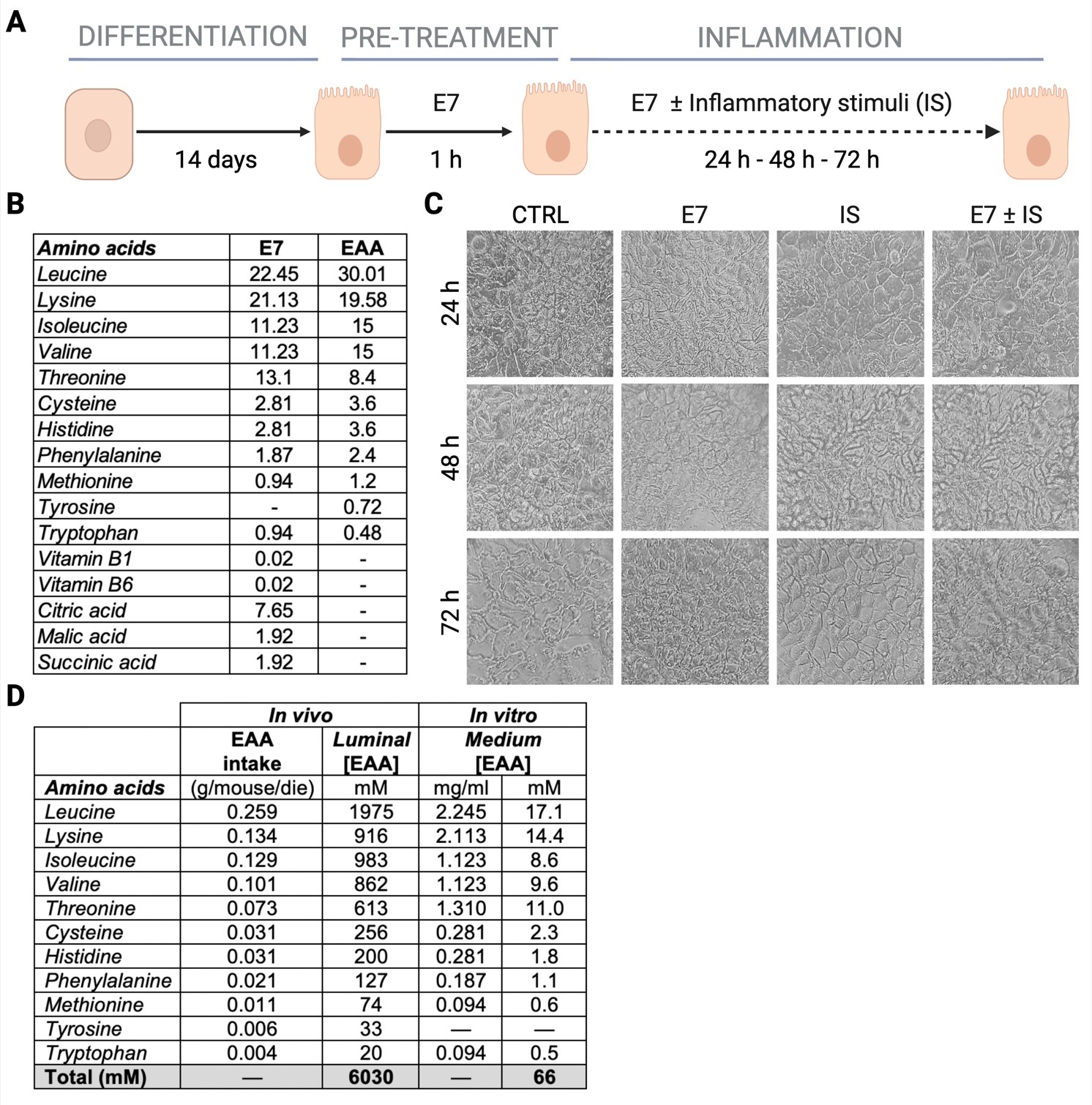


**Supplementary Figure 2. *In vitro* experimental design and amino acid composition.** (A) Schematic representation of the *in vitro* gut inflammation model (created with BioRender). (B) Composition of amino acid mixtures: E7 (essential amino acids enriched with Krebs cycle intermediates) and EAA (essential amino acids without Krebs cycle intermediates). Values are expressed as percentage (g/100 g of mixture). (C) Representative image of Caco-2 cells differentiated for 14 days and treated with E7 (1.0 %, w/v), with or without inflammatory stimuli (IS), for 24, 48, or 72 h (magnification: 20x); (D) Comparison of EAA concentrations *in vivo* and *in vitro*. *EAA intake* (g/mouse/day): daily intake of each amino acid per mouse fed with HFD-EAA (FI = 4.42 g/mouse/day). *Luminal* *[EAA] (mM)*: estimated amino acid concentration in 1 mL of intestinal lumen immediately post-ingestion. *Medium* *[EAA]*: amino acid concentration in 1 mL of culture medium of Caco-2 cells treated with E7 1% (10 mg/mL), expressed in mg/mL and mM.

**
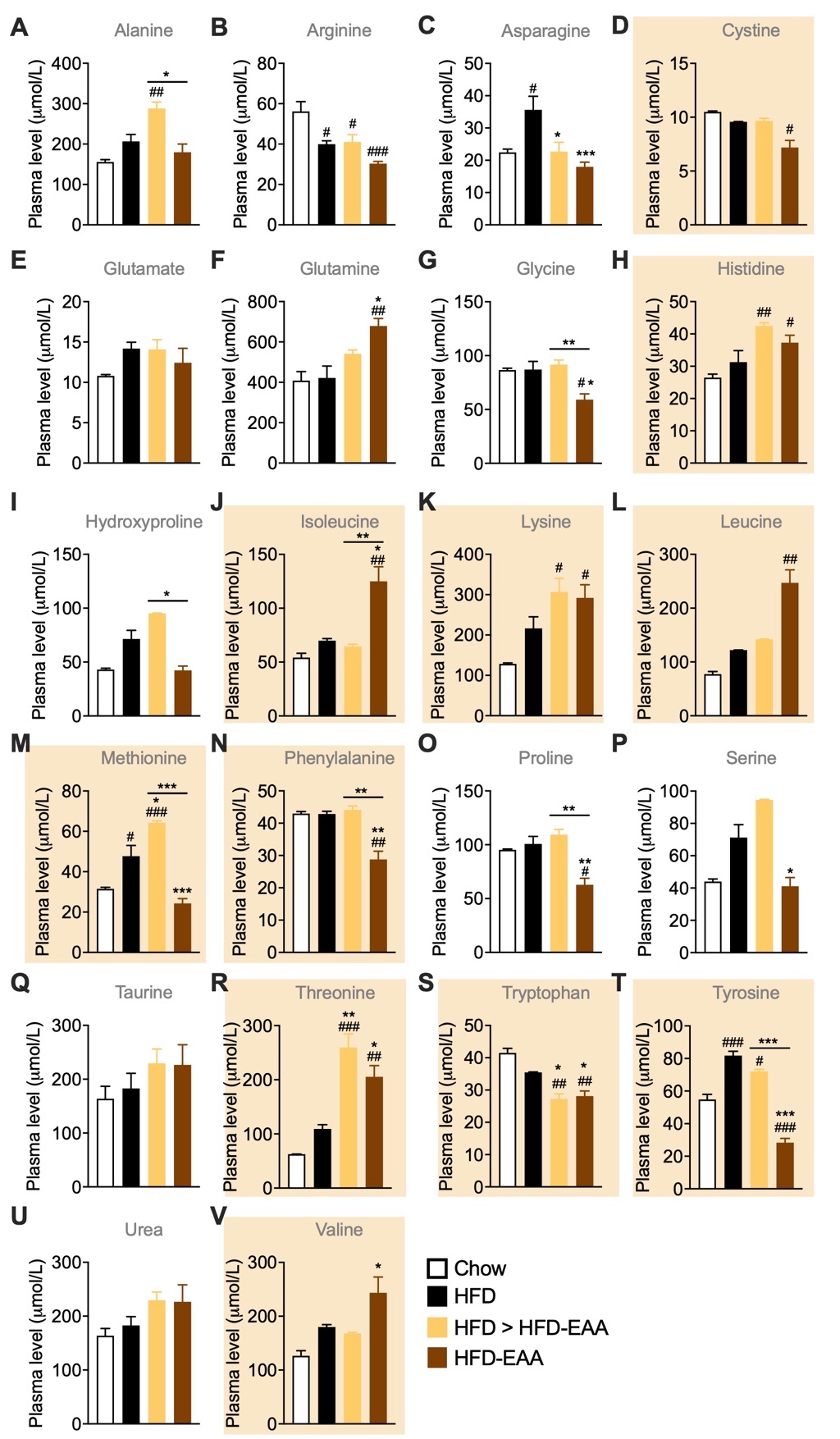
**

**Supplementary Figure 3. HFD-EAA selectively modulates circulating amino acid levels in mice.** Plasma concentrations of amino acids (µmol/L) in mice fed HFD, HFD-EAA, or chow diet for 33 weeks. Yellow-highlighted boxes indicate amino acids included in the HFD-EAA formulation. Data are mean ± SEM (n = 3-6/group). One-way ANOVA (A-D, F-H, J, K, M-O, R-T) or Kruskal–Wallis test (E, I, L, P, Q, U, V). #p < 0.05, ##p < 0.01, ###p < 0.001 vs. Chow; *p < 0.05, **p < 0.01 vs. HFD.

**
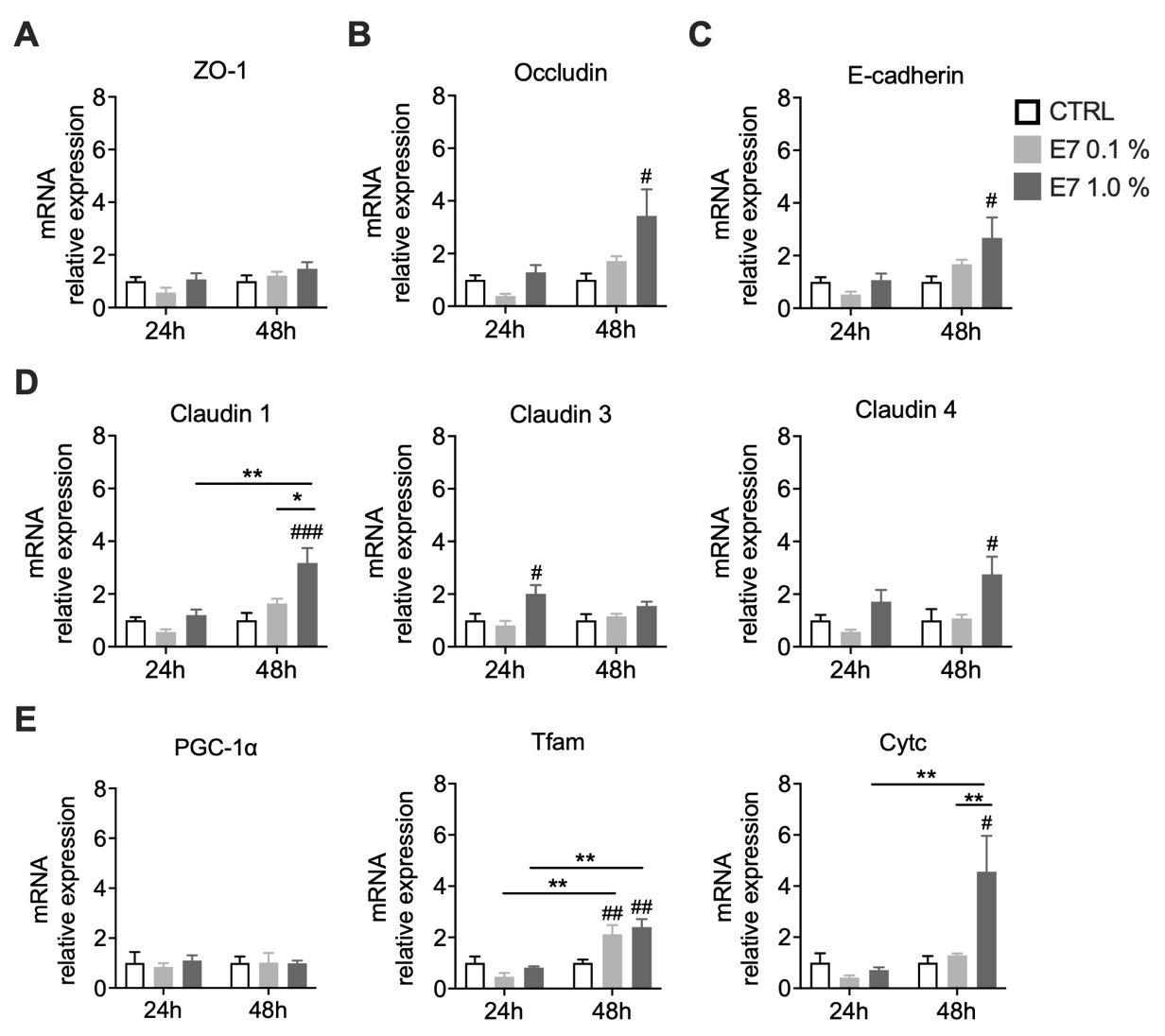
**

**Supplementary Figure 4. Effect of E7 on gut permeability and mitochondrial gene expression in Caco-2 cells after 21 days of differentiation.** (A-D) mRNA levels of intestinal permeability genes. (E) mRNA levels of mitochondrial biogenesis genes. Caco-2 cells differentiated for 21 days were treated with E7 (0.1 or 1.0 %) for 24 or 48 h. Transcript levels were normalized to GAPDH and expressed relative to untreated control (CTRL) cells (set as 1.0). Data are mean ± SEM (n = 4/group). Two-way ANOVA. #p<0.05, ##p<0.01, ###p<0.001 vs. CTRL.

**
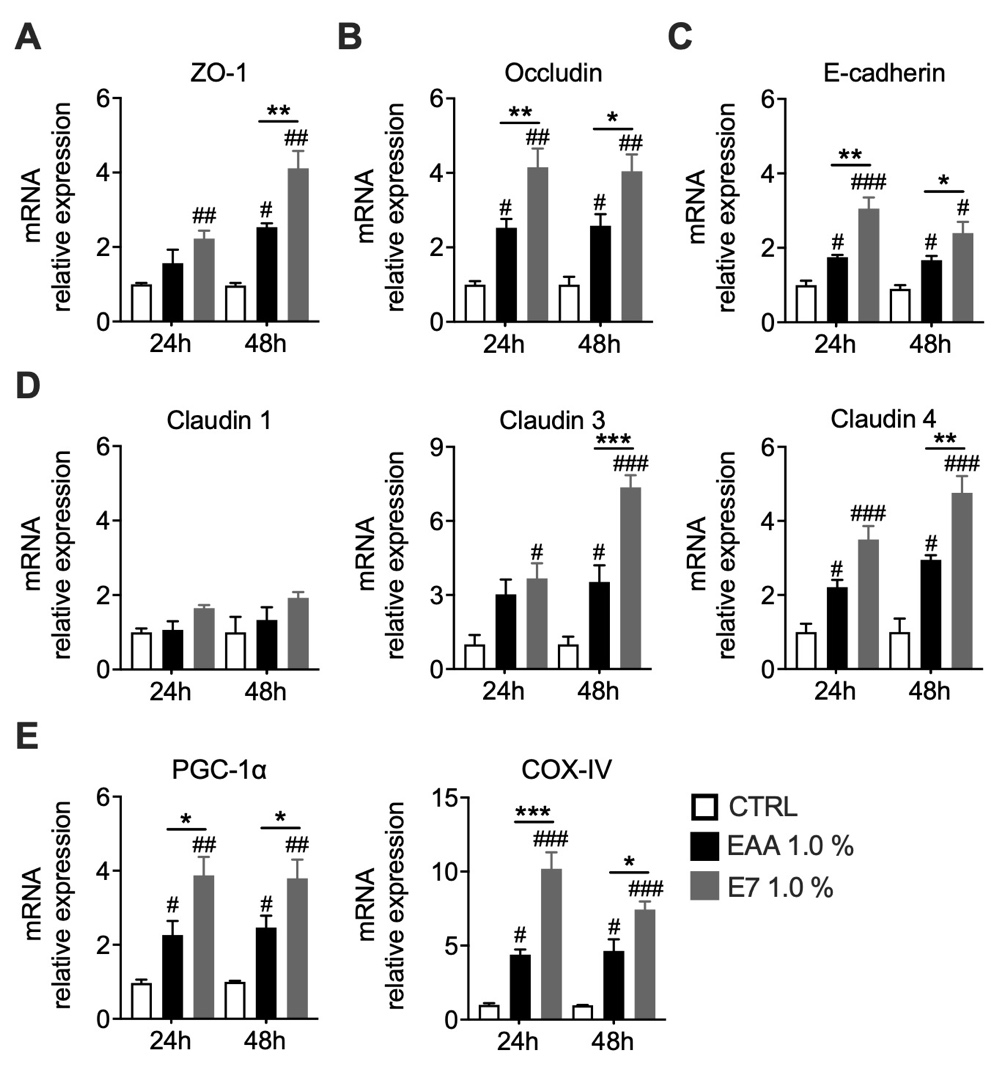
**

**Supplementary Figure 5. Comparative effects of EAA and E7 mixtures on Caco-2 cell permeability and mitochondrial gene expression** (A-D) mRNA levels of intestinal permeability genes. (E) mRNA levels of mitochondrial biogenesis genes. Caco-2 cells differentiated for 14 days were treated with EAA or E7 (both 1.0 %) for 24 or 48 h. Transcript levels were normalized to GAPDH and expressed relative to untreated control (CTRL) cells (set as 1.0). Data are mean ± SEM (n = 3-6/group). Two-way ANOVA. #p<0.05, ##p<0.01, ###p<0.001 vs. CTRL.


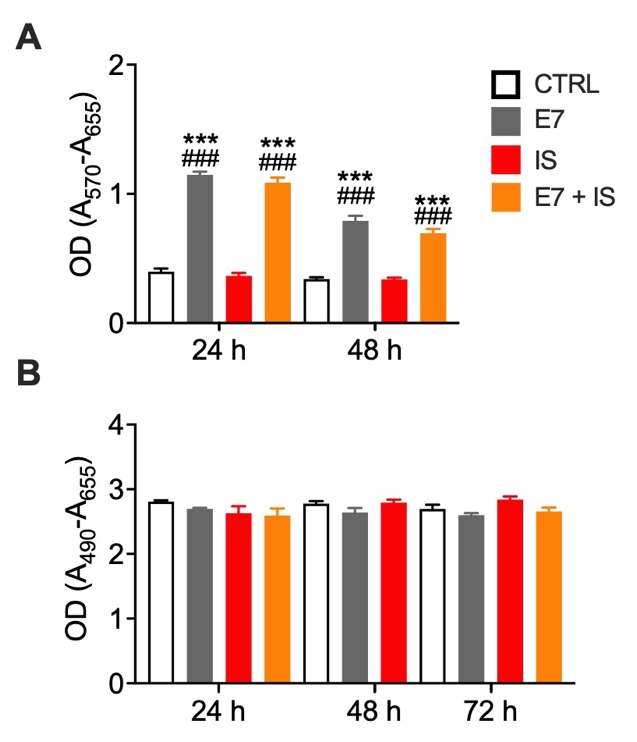


**Supplementary Figure 6.** **Impact of E7 and IS on cell viability in an *in vitro* model of gut inflammation.** Caco-2 cells differentiated for 14 days were treated with E7 (1.0 %) ± inflammatory stimuli [IL-1β (25 ng/ml), TNF-α (50 ng/ml), and LPS (10 μg/ml); IS] for 24, 48, or 72 h. (A, B) Viability measured by MTT assay (A) and TOX6 assay (B). Data are mean ± SEM (n = 4 per group). Two-way ANOVA, ###p<0.001 vs. CTRL; ***p<0.001 vs. IS.

**Supplementary Table 1 – Primer sequences for qRT-PCR**

| **Gene** | **Primer Sense** | **Primer Antisense** | **T_a_** |
| --- | --- | --- | --- |
| *Hs Claudin 1* | CCGTTGGCATGAAGTGTATG | CCAGTGAAGAGAGCCTGACC | 60 |
| *Hs Claudin 3* | AAGGTGTACGACTCGCTGCT | AGTCCCGGATAATGGTGTTG | 60 |
| *Hs Claudin 4* | GTCTGCCTGCATCTCCTCTGT | CCTCTAAACCCGTCCATCCA | 60 |
| *Hs Catalase* | ATACCTGTGAACTGTCCCTACC | GTTGAATCTCCGCACTTCTCC | 60 |
| *Hs COX-IV* | CCAGAAGGCATTGAAGGAGA | GGGCCGTACACATAGTGCTT | 60 |
| *Hs Cyt c* | CGTTGTGCCAGCGACTAAAAA | TTCCGCCCAAAGAGACCA | 60 |
| *Hs E-cadherin* | TGGACCGAGAGAGTTTCCCT | CCCTTGTACGTGGTGGGATT | 60 |
| *Hs GAPDH* | GGCTGAGAACGGGAAGCTTGT | CCAGCATCGCCCCACTTGAT | 60 |
| *Hs Occludin* | GAAGCCAAACCTCTGTGAGC | GAAGACATCGTCTGGGGTGT | 60 |
| *Hs PGC-1⍺* | AGCTGCTGAAGAGGCAAGAG | TTCCCCTAAACCAAGCACAC | 60 |
| *Hs SOD1* | GAGACGGGGTGCTGGTTTGC | ACGCCGAGGTCCTGGTTCC | 60 |
| *Hs SOD2* | TAACGGTGGTGGAGAACCCAAA | TTGAAACCAAGCCAACCCCAAC | 60 |
| *Hs Tfam* | AGATTGGGGTCGGGTCAC | GACAACTTGCCAAGACAGATG | 60 |
| *Hs ZO-1* | GAATGATGGTTGGTATGGTGCG | TCAGAAGTGTGTCTACTGTCCG | 60 |
| *Mm Claudin 1* | GCTGCTGGGTTTCATCCTGGC | GGTCACGATGTTGTCCCCAGC | 60 |
| *Mm Claudin 15* | GGACTCCGCTGCACCAACG | CCACCATCCCACAGGCTCCA | 60 |
| *Mm Claudin 2* | TCGGGACTTCTACTCGCCGC | CTGGGGCGAGCAGGAAAAGC | 60 |
| *Mm Claudin 3* | GGGCCTGTGGATGAACTGCG | TGGACACCACGATGAGGGCT | 60 |
| *Mm Claudin 4* | CCGTGTTCATCGTGGCAAGCA | TGCCCGGAAGCCACCATAGG | 60 |
| *Mm Claudin 7* | CCCCTTGACGCCCATGAACG | GCTTTGCTTTCAGTGCCTGGACA | 60 |
| *Mm COX-IV* | TGGGACTATGACAAGAATGAGTGG | TTAGCATGGACCATTGGATACGG | 63 |
| *Mm E-cadherin* | CGGGAATCGTGGCAGCAGGA | TGACCACCGTTCTCCTCCGT | 60 |
| *Mm eNOS* | CCTTCCGCTACCAGCCAGA | CAGAGATCTTCACTGCATTGGCTA | 61 |
| *Mm GAPDH* | AACTTTGGCATTGTGGAAGG | ACACATTGGGGGTAGGAACA | 60 |
| *Mm IL6* | TACCACTCCCAACAGACCTG | ACTCCAGAAGACCAGAGGAA | 60 |
| *Mm IL8* | CTGGCCGTGGCTCTCTTG | GGGTGGAAAGGTTTGGAGTATG | 60 |
| *Mm LAT1* | AAAGGCTGCGACCCGTGTGC | TGCAGGTTGGACGCATCACCT | 60 |
| *Mm NRF1* | ACAGATAGTCCTGTCTGGGGAAA | TGGTACATGCTCACAGGGATCT | 61 |
| *Mm Occludin* | GCAGGAAAGGGCAAGAGGACG | CCCTGACCCAGTCCTCCTCC | 60 |
| *Mm PGC-1α* | ACTATGAATCAAGCCACTACAGAC | TTCATCCCTCTTGAGCCTTTCG | 61 |
| *Mm SLC25A44* | CCCGTGTGCAGGTTGAAGGC | ACGATGGTGGAGGGTGTAGCC | 60 |
| *Mm Tfam* | AAGACCTCGTTCAGCATATAACATT | TTTTCCAAGCCTCATTTACAAGC | 60 |
| *Mm TNFα* | ACCACGCTCTTCTGTCTACT | TCCACTTGGTGGTTTGCTAC | 60 |
| *Mm ZO-1* | CGCTCTTCCTCTCTGCTCCGG | GCCGCAGCCAGTCACGATCT | 60 |

T_a_, temperature of annealing (°C). *Mm*, *mus musculus; Hs, homo sapiens*. *COX-IV*, cytochrome c oxidase subunit IV; *eNOS*, endothelial nitric oxide synthase; *GAPDH*, glyceraldehyde 3-phosphate dehydrogenase; Interleukin (*IL) 6* and *8*; *LAT1*, L-type amino acid transporter 1 (*SLC7A5*); *NRF1*, Nuclear Respiratory Factor 1; *PGC-1α*, peroxisome proliferator-activated receptor γ coactivator 1α; *SLC25A44*, solute carrier family 25 member 44 (mitochondrial); SOD, Superoxide dismutase 1 (cytoplasmatic) and 2 (mitochondrial); *Tfam*, transcription factor A; *TNFα*, tumor necrosis factor α; *ZO-1*, zonulin 1.

**Supplementary Table 2 — Pearson’s correlation analysis**

|  | **BW** | | **Glucose homeostasis** | | **eWAT weight** | | **Intestine**  **lenght** | | **Intestine**  **weightcs** | | **Citrulline**  **plasma level** | | **Calprotectin**  **plasma level** | | **Faeces**  **excrection** | | **Food**  **excreted** | |
| --- | --- | --- | --- | --- | --- | --- | --- | --- | --- | --- | --- | --- | --- | --- | --- | --- | --- | --- |
| **Plasma**  **amino acid** | *r* | *p* | *r* | *p* | *r* | *p* | *r* | *p* | *r* | *p* | *r* | *p* | *r* | *p* | *r* | *p* | *r* | *p* |
| Alanine | 0.17 | 0.59 | 0.29 | 0.36 | 0.04 | 0.91 | -0.11 | 0.74 | 0.10 | 0.76 | -0.03 | 0.93 | 0.03 | 0.92 | -0.32 | 0.31 | -0.19 | 0.56 |
| **Arginine** | -0.44 | 0.15 | 0.38 | 0.22 | 0.09 | 0.79 | 0.27 | 0.39 | 0.80 | 0.00 | -0.02 | 0.96 | -0.22 | 0.48 | **0.62** | **0.03** | **0.62** | **0.03** |
| **Asparagine** | 0.27 | 0.40 | **0.59** | **0.04** | **0.72** | **0.01** | 0.05 | 0.88 | 0.02 | 0.94 | -0.03 | 0.92 | -0.16 | 0.61 | -0.21 | 0.52 | -0.17 | 0.60 |
| **Cystine** | -0.19 | 0.56 | 0.48 | 0.11 | 0.21 | 0.51 | -0.08 | 0.81 | **0.64** | **0.02** | -0.15 | 0.65 | -0.06 | 0.85 | 0.41 | 0.19 | 0.36 | 0.25 |
| Glutamate | 0.48 | 0.11 | 0.15 | 0.65 | 0.19 | 0.55 | 0.20 | 0.54 | -0.08 | 0.81 | -0.16 | 0.62 | -0.13 | 0.70 | -0.30 | 0.35 | -0.12 | 0.71 |
| Glutamine | -0.08 | 0.80 | -0.42 | 0.17 | -0.43 | 0.16 | -0.13 | 0.69 | -0.51 | 0.09 | 0.46 | 0.14 | 0.07 | 0.84 | -0.42 | 0.18 | -0.23 | 0.46 |
| Glycine | -0.21 | 0.51 | 0.34 | 0.27 | 0.11 | 0.72 | -0.07 | 0.83 | 0.45 | 0.14 | -0.24 | 0.45 | -0.47 | 0.12 | 0.23 | 0.47 | 0.21 | 0.50 |
| **Histidine** | 0.07 | 0.83 | 0.05 | 0.88 | -0.09 | 0.79 | -0.07 | 0.82 | -0.33 | 0.30 | 0.11 | 0.74 | -0.05 | 0.87 | **-0.58** | **0.05** | -0.45 | 0.15 |
| Hydroxyproline | 0.16 | 0.61 | 0.39 | 0.22 | 0.26 | 0.41 | -0.31 | 0.33 | 0.14 | 0.66 | -0.37 | 0.24 | -0.09 | 0.78 | -0.27 | 0.39 | -0.33 | 0.29 |
| **Isoleucine** | -0.06 | 0.86 | 0.06 | 0.86 | -0.14 | 0.66 | 0.21 | 0.50 | -0.40 | 0.20 | **0.61** | **0.03** | 0.23 | 0.47 | -0.37 | 0.23 | -0.02 | 0.94 |
| **Leucine** | 0.01 | 0.98 | -0.07 | 0.83 | -0.23 | 0.48 | 0.10 | 0.76 | -0.45 | 0.15 | **0.60** | **0.04** | 0.40 | 0.20 | -0.45 | 0.15 | -0.15 | 0.64 |
| **Lysine** | -0.05 | 0.88 | 0.07 | 0.83 | -0.04 | 0.89 | -0.06 | 0.85 | -0.27 | 0.39 | 0.41 | 0.19 | 0.31 | 0.33 | **-0.59** | **0.04** | -0.44 | 0.15 |
| Methionine | 0.12 | 0.70 | 0.43 | 0.16 | 0.28 | 0.39 | -0.28 | 0.38 | 0.31 | 0.32 | -0.26 | 0.41 | 0.05 | 0.89 | -0.14 | 0.67 | -0.17 | 0.59 |
| **Phenylalanine** | -0.01 | 0.96 | **0.61** | **0.03** | 0.31 | 0.33 | -0.01 | 0.98 | 0.44 | 0.15 | -0.23 | 0.46 | -0.24 | 0.46 | 0.24 | 0.46 | 0.32 | 0.32 |
| Proline | -0.08 | 0.81 | 0.37 | 0.23 | 0.30 | 0.35 | -0.07 | 0.84 | 0.42 | 0.17 | -0.43 | 0.16 | -0.42 | 0.17 | 0.13 | 0.68 | 0.03 | 0.93 |
| Serine | 0.06 | 0.86 | 0.34 | 0.28 | 0.27 | 0.40 | -0.32 | 0.31 | 0.20 | 0.53 | -0.38 | 0.23 | -0.07 | 0.83 | -0.18 | 0.58 | -0.31 | 0.33 |
| Taurine | 0.26 | 0.42 | -0.06 | 0.84 | -0.17 | 0.59 | -0.02 | 0.96 | -0.15 | 0.63 | 0.24 | 0.46 | 0.10 | 0.76 | -0.27 | 0.39 | -0.07 | 0.84 |
| Threonine | -0.19 | 0.55 | -0.11 | 0.74 | -0.26 | 0.41 | -0.19 | 0.56 | 0.00 | 1.00 | 0.19 | 0.56 | 0.43 | 0.16 | -0.36 | 0.26 | -0.40 | 0.19 |
| **Tryptophan** | -0.25 | 0.44 | 0.26 | 0.41 | 0.24 | 0.46 | 0.33 | 0.29 | 0.54 | 0.07 | -0.02 | 0.96 | -0.34 | 0.28 | **0.59** | **0.04** | **0.61** | **0.03** |
| **Tyrosine** | 0.27 | 0.40 | **0.70** | **0.01** | **0.61** | **0.03** | -0.07 | 0.82 | 0.32 | 0.31 | -0.44 | 0.15 | -0.14 | 0.66 | -0.01 | 0.98 | -0.05 | 0.87 |
| Urea | 0.21 | 0.50 | 0.48 | 0.11 | 0.70 | 0.11 | 0.04 | 0.90 | 0.13 | 0.70 | 0.06 | 0.85 | -0.02 | 0.95 | -0.05 | 0.89 | -0.02 | 0.95 |
| **Valine** | -0.05 | 0.88 | 0.01 | 0.99 | -0.10 | 0.75 | 0.13 | 0.69 | -0.40 | 0.19 | **0.70** | **0.01** | 0.52 | 0.08 | -0.42 | 0.17 | -0.14 | 0.66 |

Amino acid plasma levels were correlated with body weight (BW), adiposity [epididymal white adipose tissue (eWAT) weight, g], glucose homeostasis [area under the curve (AUC) from glucose tolerance test], intestinal length (cm) and weight (g/BW), plasma calprotectin (pg/ml) and cintrulline (µmol/L) levels, feaecal excrection (g), and food percentage of food excreted (%). Pearson correlation coefficient (r) and *p* values were reported; positive correlations are indicated in red, negative correlations in blue.

**Uncropped blots**

**Figure 4 Panel F and Figure 5 Panel B Vinculin WB full scan of the entire original gel**

**
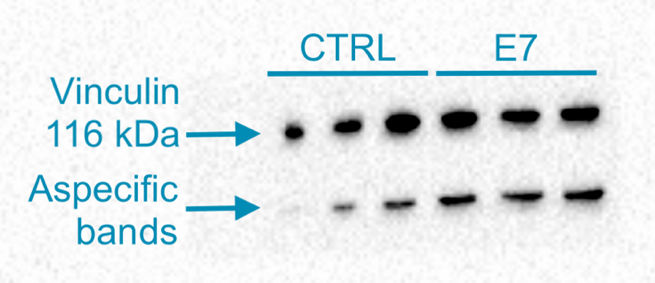
**

**Figure 4 Panel F OCCLUDIN WB full scan of the entire original gel**

**
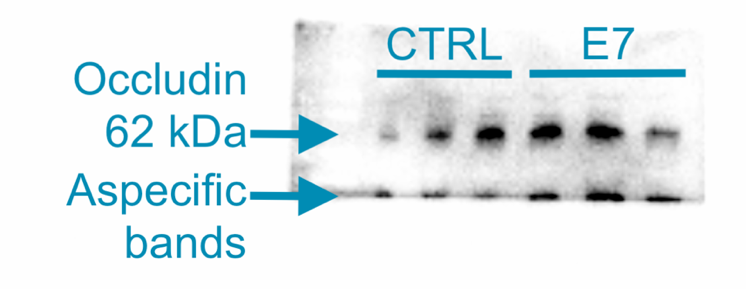
**

**Figure 4 Panel F ZO-1 WB full scan of the entire original gel**

**
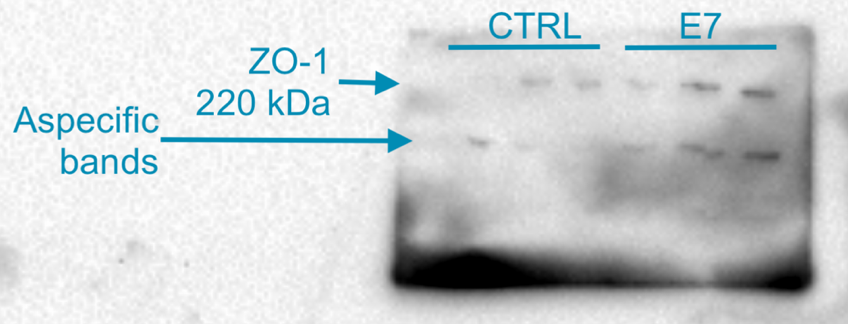
**

**Figure 5 Panel B COXIV WB full scan of the entire original gel**

**
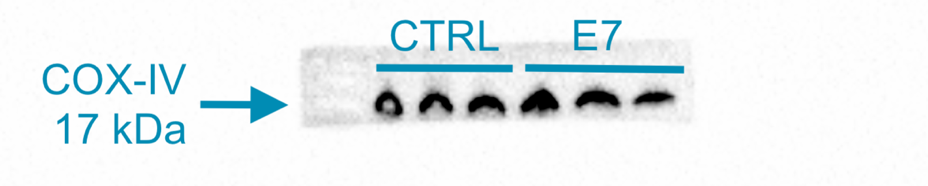
**

**Figure 5 Panel B PGC1a WB full scan of the entire original gel**


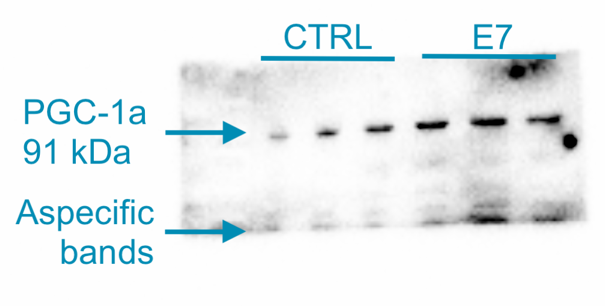


**Figure 5 Panel D p-eNOS WB full scan of the entire original gel**

**
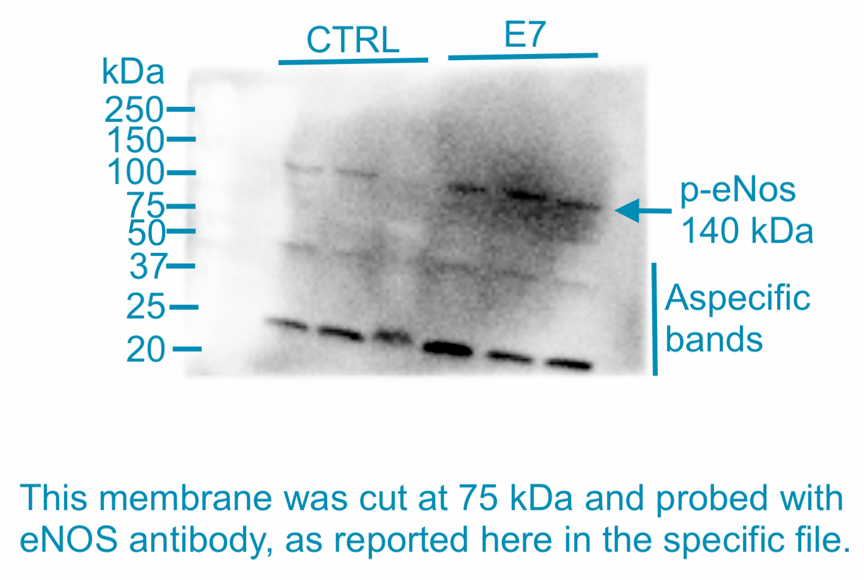
**

**Figure 5 Panel D eNOS WB full scan of the entire original gel**


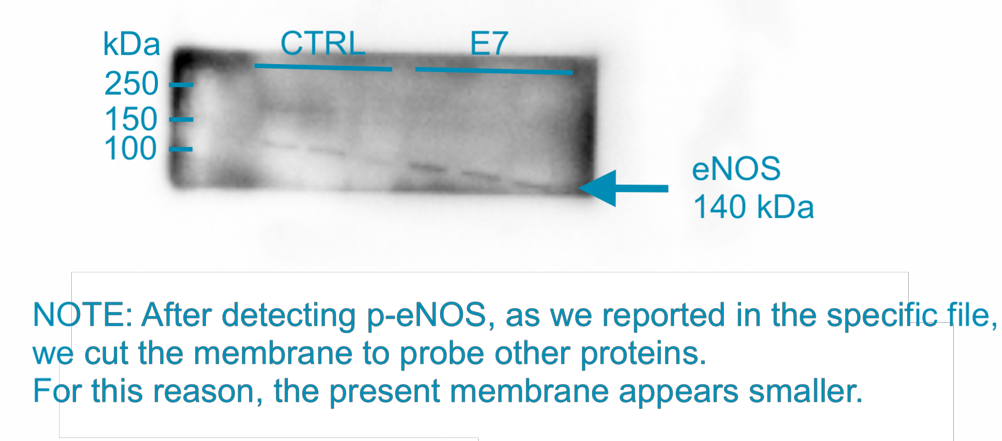


**Figure 5 Panel D Vinculin WB full scan of the entire original gel**

**
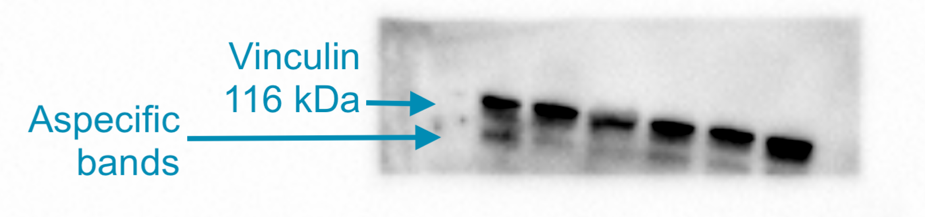
**

**Figure 5 Panel E p-p70S6K WB full scan of the entire original gel**

**
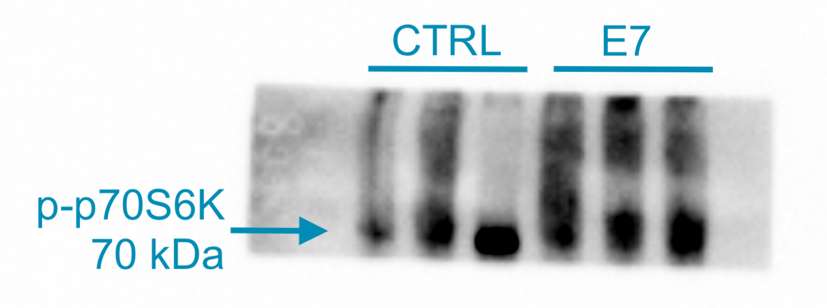
**

**Figure 5 Panel E p70S6K WB full scan of the entire original gel**

**
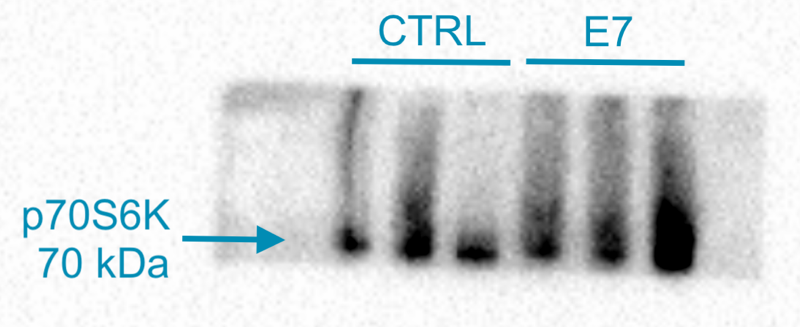
**

**Figure 5 Panel E pS6 WB full scan of the entire original gel**

**
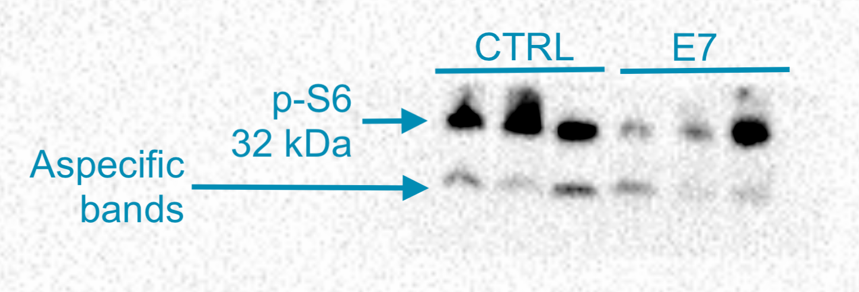
**

**Figure 5 Panel E S6 WB full scan of the entire original gel**

**
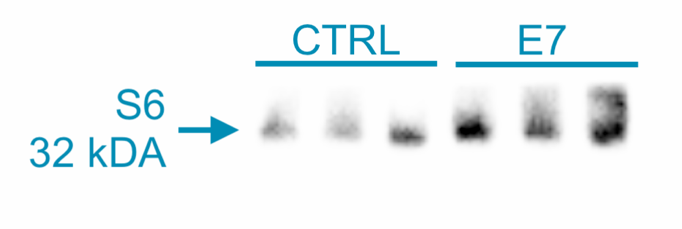
**

**Figure 5 Panel E Vinculin WB full scan of the entire original gel**

**
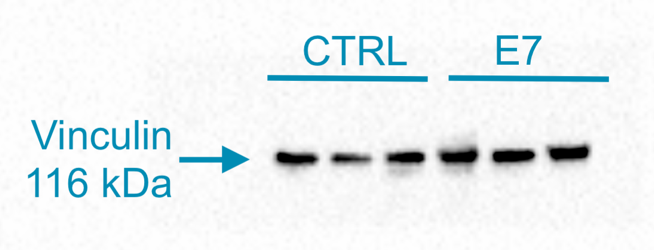
**

**Figure 7 Panel B COXIV WB full scan of the entire original gel**

**
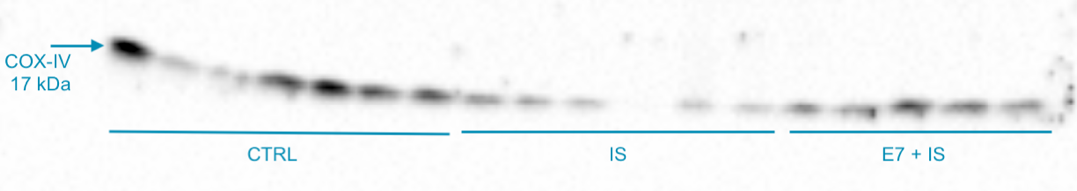
**

**Figure 7 Panel B PGC1a WB full scan of the entire original gel**

**
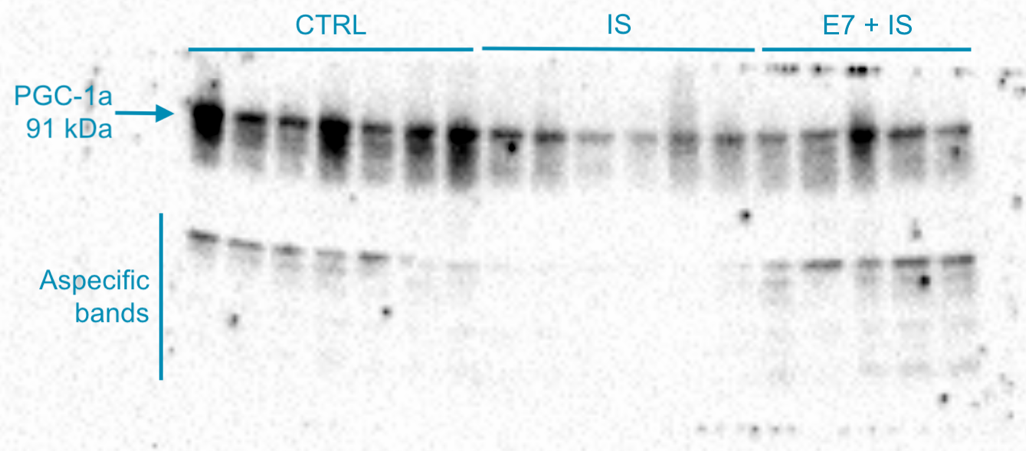
**

**Figure 7 Panel B Vinculin WB full scan of the entire original gel**

**
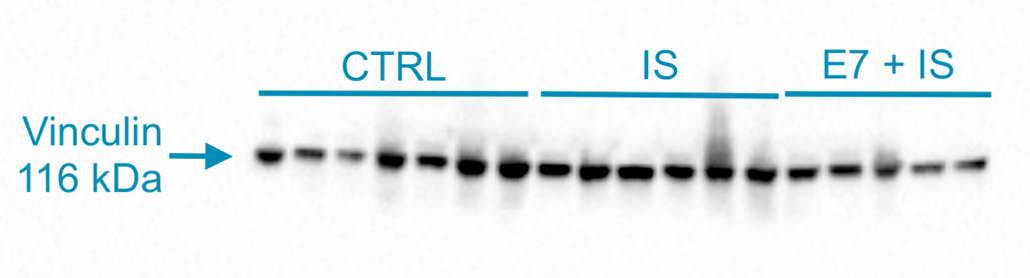
**

**Figure 7 Panel H PGC1a WB full scan of the entire original gel**

**
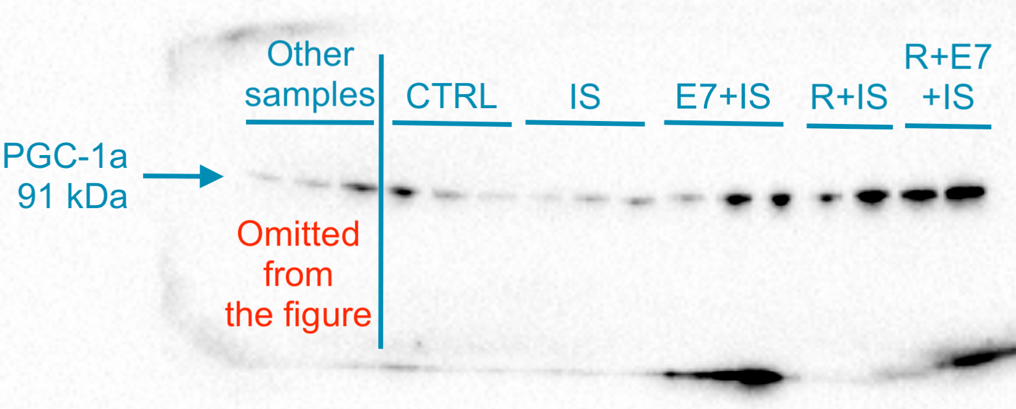
**

**Figure 7 Panel H ZO1 WB full scan of the entire original gel**

**
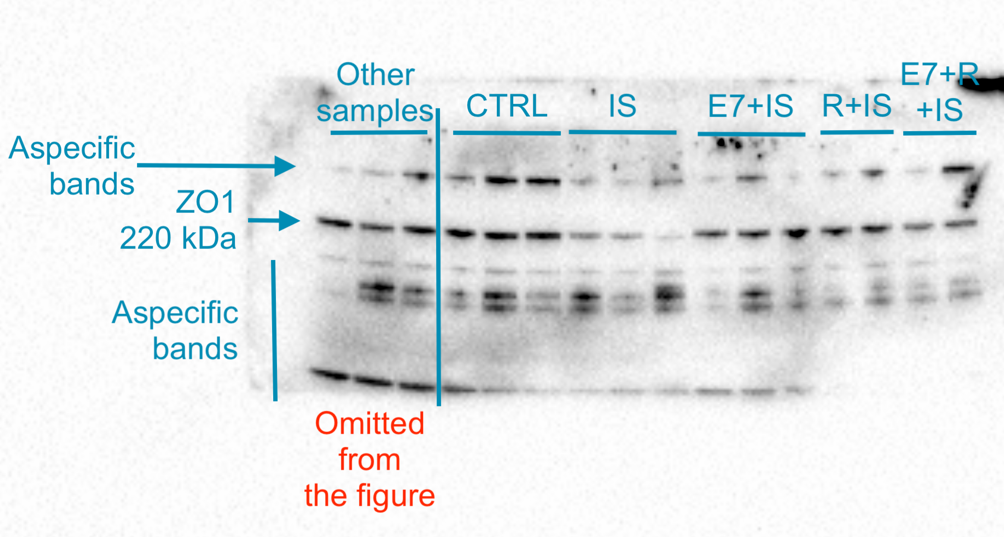
**

**Figure 7 Panel H Vinculin WB full scan of the entire original gel**

**
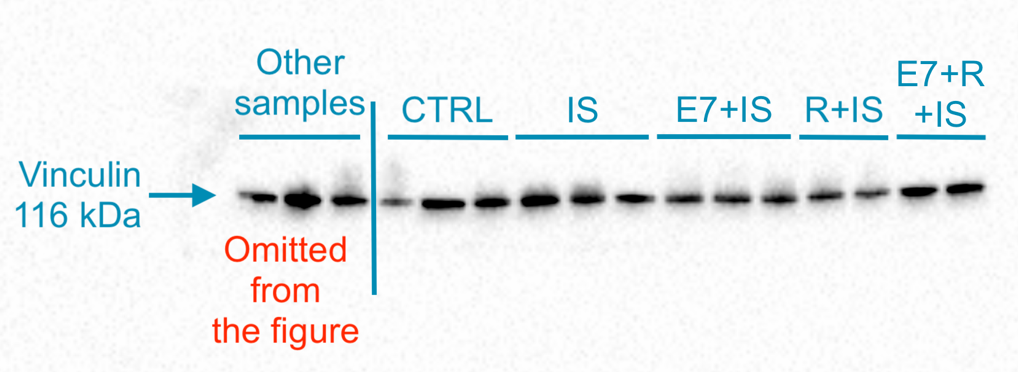
**
